# Spontaneous Pneumothorax: A Review of Underlying Etiologies and Diagnostic Imaging Modalities

**DOI:** 10.3390/tomography11110125

**Published:** 2025-11-07

**Authors:** Rupali Jain, Vinay Kandula, Drew A. Torigian, Achala Donuru

**Affiliations:** 1 Hospital of University of Pennsylvania, Philadelphia, PA 19104, USA; rupali.jain@pennmedicine.upenn.edu (R.J.); vkandula@nemours.org (V.K.); drew.torigian@pennmedicine.upenn.edu (D.A.T.); 2 Nemours Alfred I. duPont Hospital for Children, Wilmington, DE 19104, USA

**Keywords:** spontaneous pneumothorax, thorax, etiologies, CT, medical imaging

## Abstract

**Simple Summary:**

A collapsed lung (spontaneous pneumothorax) can happen without a clear cause, but it is often the first sign of an underlying, sometimes hidden, lung disease. This review serves as a comprehensive visual guide for doctors to help them recognize the wide variety of conditions that can lead to a collapsed lung. We show that these causes are incredibly diverse, ranging from common conditions like chronic obstructive pulmonary disease (COPD) and infections to rarer genetic disorders and cancers. The review emphasizes that while a chest X-ray is often the first step, a CT scan is the most powerful tool for uncovering the specific underlying problem. Knowing the exact cause is critical for choosing the right treatment, predicting if the lung might collapse again, and improving a patient’s overall outcome.

**Abstract:**

This review focuses on the diverse etiologies of secondary spontaneous pneumothorax (SSP) and the crucial role of imaging in their diagnosis. Unlike primary spontaneous pneumothorax (PSP), which is typically due to ruptured blebs, SSP results from a wide array of underlying pulmonary conditions that can pose significant diagnostic challenges. These include infections like tuberculosis, airway diseases such as chronic obstructive pulmonary disease, malignancies (primary and metastatic), interstitial lung diseases like sarcoidosis, cystic lung diseases such as lymphangioleiomyomatosis, and connective tissue disorders. In women, catamenial pneumothorax secondary to endometriosis should be considered. The role of radiologists is crucial in uncovering these underlying conditions. While chest radiography is the initial imaging modality, computed tomography (CT) provides superior sensitivity for detecting subtle parenchymal abnormalities. Advanced techniques like photon-counting detector CT offer further benefits, including enhanced spatial resolution, reduced noise, and lower radiation dose, potentially revealing underlying causes that might be missed with conventional CT. This enhanced visualization of subtle parenchymal changes, small airways, and vascular structures can be the key to diagnosing the underlying cause of pneumothorax. Recognizing the diverse etiologies of SSP and utilizing advanced imaging techniques is paramount for accurate diagnosis, appropriate management, and improved patient outcomes.

## 1. Introduction

**Figure 1:**
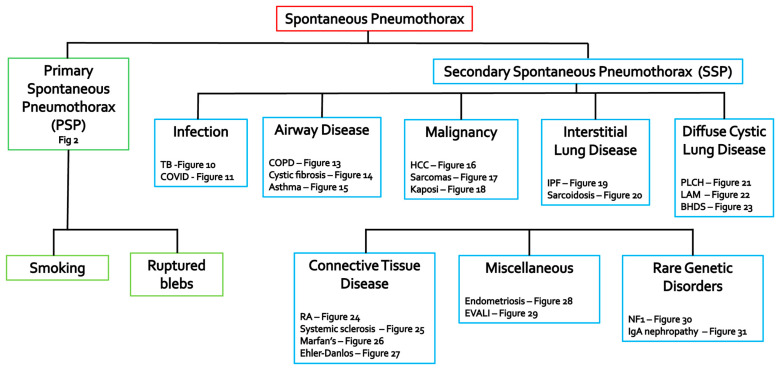
Etiological roadmap of spontaneous pneumothorax. Diagram illustrating the major categories and specific conditions leading to primary spontaneous pneumothorax (PSP) and secondary spontaneous pneumothorax (SSP). Key etiologies of SSP are grouped into infection, airway disease, malignancy, interstitial lung disease, diffuse cystic lung disease, connective tissue disease, miscellaneous causes, and rare genetic disorders, with corresponding illustrative figure numbers from the manuscript indicated for each condition.

Pneumothorax signifies intrapleural gas accumulation between the visceral and parietal layers. It can be broadly classified as traumatic/iatrogenic or spontaneous. This review will focus on spontaneous pneumothorax (SP), which is further subdivided into two distinct categories. Primary spontaneous pneumothorax (PSP) occurs without any clinically apparent underlying lung disease, whereas secondary spontaneous pneumothorax (SSP) develops as a complication of pre-existing pulmonary conditions. While PSP is a well-defined clinical entity, the causes of SSP are numerous and varied, often posing a diagnostic challenge. The purpose of this pictorial review is to provide a comprehensive overview of the diverse etiologies of spontaneous pneumothorax, with a particular focus on SSP, and to highlight the crucial role of modern diagnostic imaging in uncovering these underlying causes. Understanding the clinical context and imaging findings is paramount, as the clinical sequelae of pneumothorax range widely from incidental detection to life-threatening circulatory collapse [1,2,3,4,5,6] (Figure 1).

The estimated annual incidence of SP in males ranges from 4.2 to 16.8 per 100,000, whereas in females it ranges from 3.8 to 9.8 per 100,000 annually, with documented geographic variability [7,8,9]. SP has a varied age distribution, with PSP predominantly occurring in adolescents and young adults (15–34 years) and SSP predominantly occurring in children or adults over 55 years of age [7,10]. SSP has a higher recurrence rate ranging from 40% to 56% as compared to 16% to 54% in PSP [11,12,13,14,15,16].

In this article, we review the categorization, etiologies, and imaging modalities for SP, with the main emphasis on familiarizing radiologists with the various disorders and associated key imaging features that may indicate potential underlying culprit etiologies for SP.

## 2. Classification and Etiologies of Spontaneous Pneumothorax

### 2.1. Primary Spontaneous Pneumothorax (PSP)

**Figure 2:**
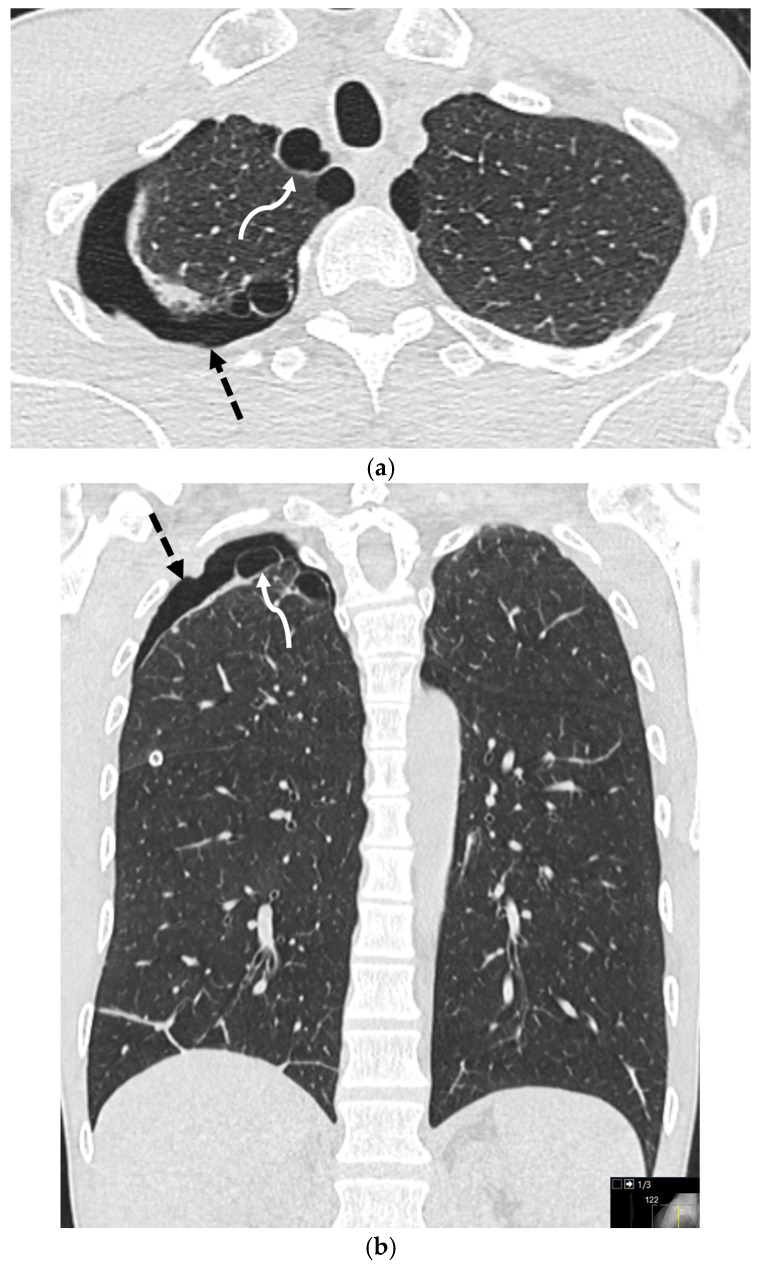
45-year-old male with no known past medical history presented to the emergency department with right-sided chest pain and dyspnea. He was found to have a large right-sided pneumothorax. Axial (**a**) and coronal (**b**) images from CT chest in lung windows demonstrate a small right pneumothorax (dashed arrows). Incidental note is made of apical pleural blebs (curved arrows).

Primary spontaneous pneumothorax (PSP) occurs without clinically apparent lung disease, though underlying apical blebs or bullae are often identified on CT. The primary risk factor is tobacco use, which can increase the risk up to twentyfold, with a taller, thinner body habitus also being a predisposing factor [17,18,19] (Figure 2).

### 2.2. Secondary Spontaneous Pneumothorax (SSP)

SSP arises from numerous underlying lung conditions, many of which are detailed in this review. When an inherited syndrome is suspected, genetic evaluation is clinically valuable. Identifying specific gene mutations can confirm the diagnosis, guide management, estimate recurrence risks, influence treatment strategies, and enable cascade screening for at-risk relatives [20]. The etiologies of SSP are vast, spanning a wide spectrum of conditions that range from common to exceedingly rare. To provide a clinical framework and address the relative risks of these underlying causes, Table 1 stratifies the key etiologies of SSP based on their approximate frequency, demographics, and recurrence risk. 

## 3. Diagnostic Imaging Modalities for Spontaneous Pneumothorax

Imaging modalities for pneumothorax (see Table 2 for summary).

### 3.1. Chest Radiography 

**Figure 3:**
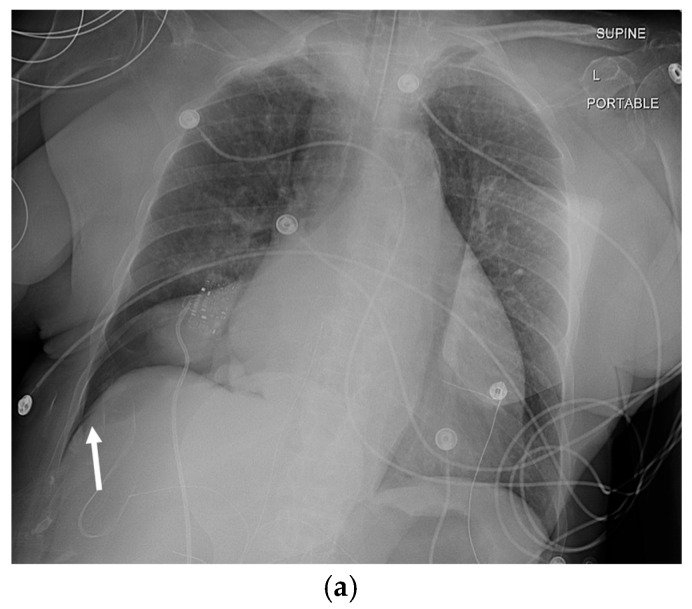

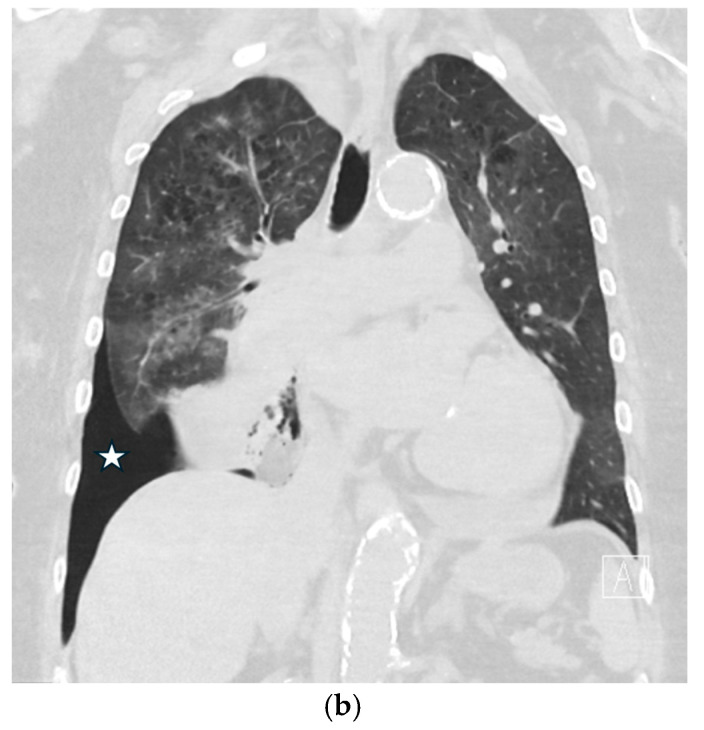
74-year-old female patient status post-cardiac arrest at home. She was resuscitated by the emergency medical services at home and arrived at the emergency department with a Glasgow coma scale of 3. The initial portable chest radiograph (**a**) demonstrated a right-sided deep sulcus sign (white arrow). Coronal image (**b**) from CT performed on the same day demonstrates a large right basilar pneumothorax (star).

**Figure 4:**
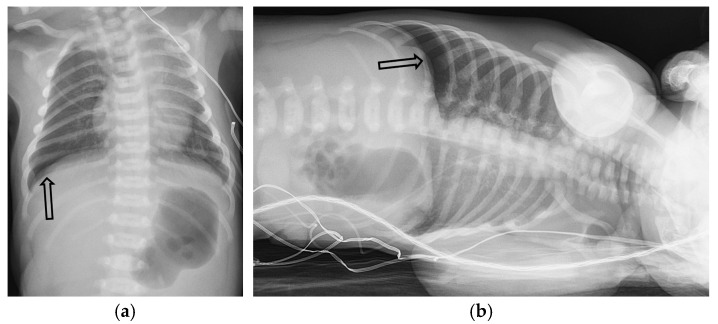
One-day-old term neonate presents with tachypnea. Frontal chest radiograph (**a**) demonstrates a small right pneumothorax (open black arrow). Left lateral decubitus frontal chest radiograph (**b**) reveals increased conspicuity of small-to-moderate right pneumothorax (open black arrow).

The key radiographic sign is a visible visceral pleural line separated from the chest wall by a lucent space without lung markings. On supine radiographs, a “deep sulcus sign” may be the only indicator. Pleural adhesions can cause loculation, requiring careful evaluation of the entire hemithorax. Supplemental views, such as expiratory or lateral decubitus images, can increase the conspicuity of small pneumothoraces [21,22] (Figure 3 and Figure 4).

### 3.2. Ultrasonography

**Figure 5:**
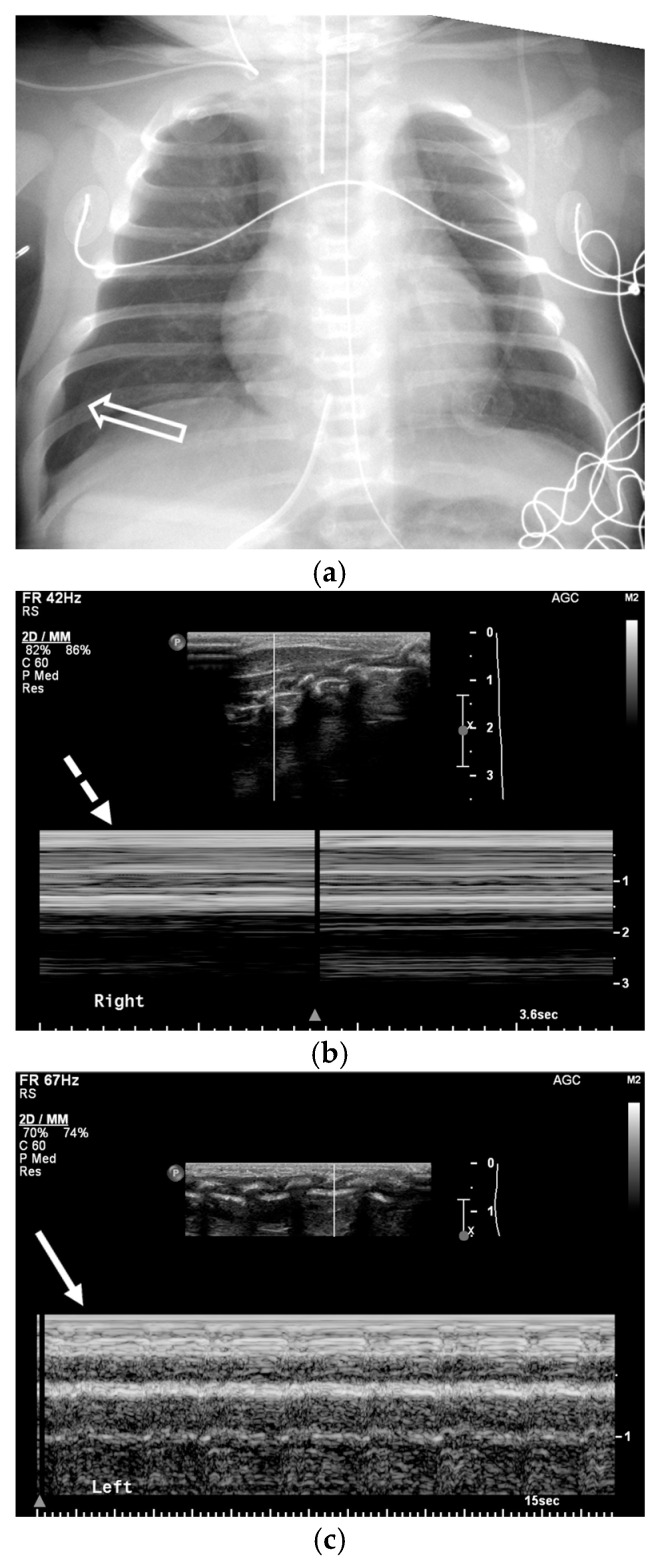
One-day-old neonate born at 39 weeks of gestation. Frontal chest radiograph (**a**) shows a small right pneumothorax (open white arrow). M-mode ultrasound image of right basilar hemithorax (**b**) reveals a barcode sign typical of pneumothorax (dashed white arrow). Comparison M-mode ultrasound image of left hemithorax (**c**) shows several normally expected B-lines and the presence of a seashore sign (solid white arrow).

In emergency and critical care settings, bedside ultrasonography offers a rapid, radiation-free method for diagnosing pneumothorax. The primary finding is the absence of “lung sliding,” which is the normal shimmering movement of the visceral pleura against the parietal pleura. On M-mode imaging, the absence of lung sliding manifests as the “barcode sign” (a series of horizontal lines), which replaces the normal “seashore sign” seen with a healthy, moving lung (Figure 5). The most specific sign for pneumothorax is the “lung point,” a transition zone on the chest wall where lung sliding disappears, representing the edge of the collapsed lung. With a reported sensitivity of over 90%, ultrasound is particularly valuable for critically ill patients or children, often outperforming supine chest radiography [22] (Figure 5).

### 3.3. Computed Tomography (CT)

CT is the definitive imaging modality for the diagnosis of SP and, crucially, for the characterization of its underlying causes.

Energy-Integrating Detector (EID)-CT: The standard protocol is a non-contrast, thin-slice (1.0–1.25 mm) acquisition reconstructed with a lung algorithm. Multiplanar reformats (MPRs) are essential and superior to radiography for delineating the visceral pleura, quantifying pneumothorax size, and detecting subtle etiologies like subpleural blebs. CT clearly distinguishes pleural gas from the confined gas of a pneumomediastinum.

**Figure 6:**
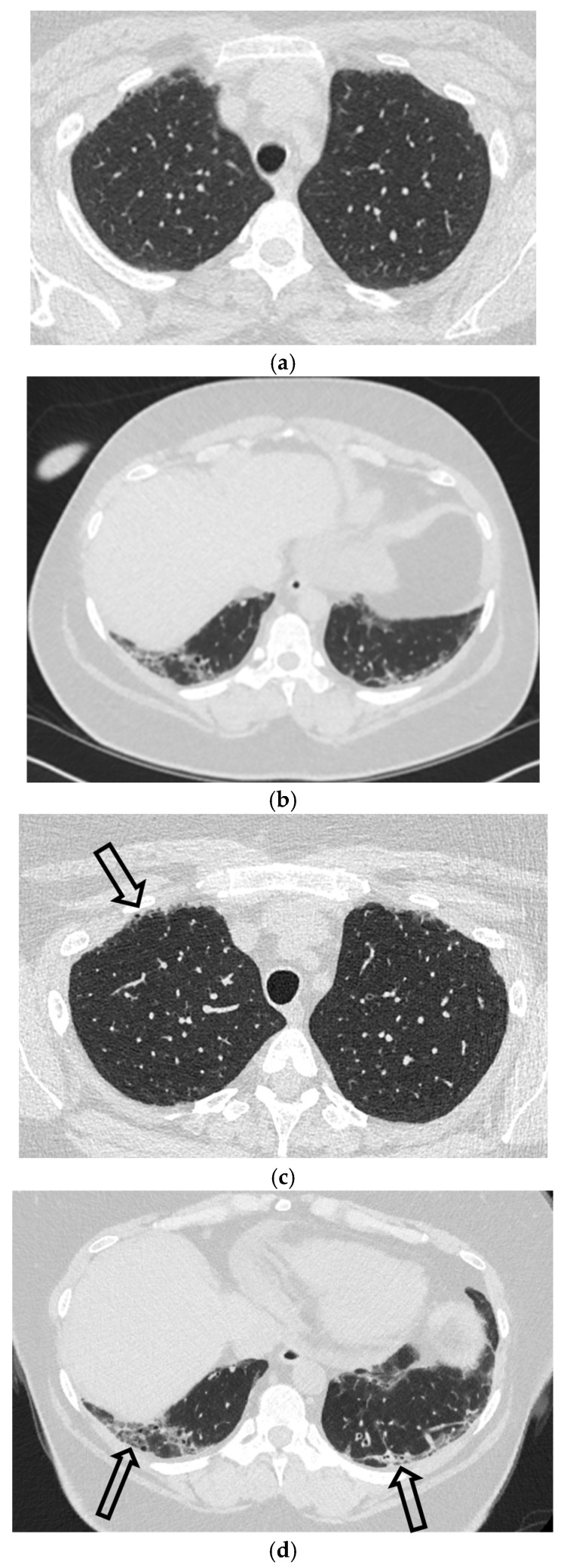
38-year-old female with systemic lupus erythematosus. Axial EID-CT images in lung window (**a**,**b**) and axial PCD-CT images in lung window obtained about 1 year later (**c**,**d**) demonstrate peripheral basilar-predominant ILD (open black arrows) consistent with NSIP. Note improved visualization of fine peripheral linear interstitial opacities, associated ground-glass opacities, and mild traction bronchiolectasis on PCD-CT images compared to EID-CT images.

Photon-Counting Detector (PCD)-CT: While EID-CT remains the standard, PCD-CT is an emerging technology offering inherently higher spatial resolution and lower image noise (Figure 6). These technical advantages, demonstrated in early studies, could theoretically improve the detection of small blebs and allow for reduced radiation dose. However, robust clinical evidence from large-scale prospective studies is still needed to determine if these improvements translate to better patient outcomes or changes in management [23,24,25].

### 3.4. Magnetic Resonance Imaging (MRI)

**Figure 7:**
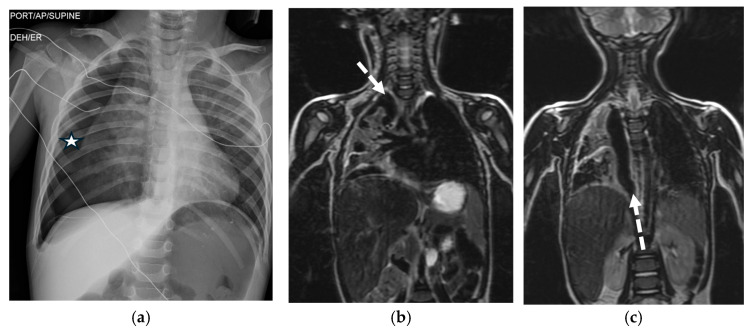
3-year-old boy presents after a motor vehicle accident. Frontal chest radiograph (**a**) demonstrates moderate-to-large right pneumothorax (white star) with associated partial atelectasis of the right lung. Coronal heavily T2W MR images obtained during thoracic spinal MRI examination (**b**,**c**) again show very-low-signal-intensity right pneumothorax (dashed white arrow) along with increased signal intensity of partially atelectatic right lung. Although MRI is not a common way to diagnose a pneumothorax, it can be used to identify one when encountered incidentally.

MRI is not typically used for the primary diagnosis of spontaneous pneumothorax (SP) because of its longer scan times, lower spatial resolution for lung tissue, and susceptibility to motion artifacts. Its main role is limited to a problem-solving tool in specific cases, such as when SP is found incidentally during a scan for other reasons (e.g., cardiac or spine evaluation), or when CT is contraindicated (e.g., in pregnancy) (Figure 7). MRI can visualize pleural surfaces and fluid without ionizing radiation. Therefore, its routine use for SP diagnosis is not recommended [21].

## 4. Pneumothorax Mimics and Diagnostic Pitfalls

**Figure 8:**
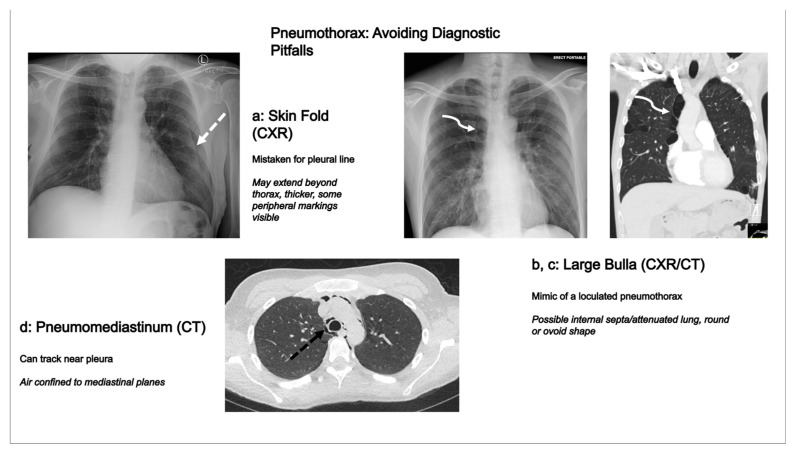
Common pneumothorax mimics and diagnostic pitfalls. (**a**) Frontal chest radiograph (CXR) demonstrating a left-sided skin fold (dashed white arrow) creating a linear lucency that can be mistaken for a visceral pleural line. Note its potential to extend beyond the thoracic cavity and the presence of faint peripheral lung markings. (**b**) Frontal CXR showing a large right apical bulla (curved white arrow) mimicking a loculated pneumothorax. (**c**) Coronal CT image in lung window further illustrating a large right apical bulla (curved white arrow) with a thin wall, which could be confused with a loculated pneumothorax. The intraparenchymal location and possible internal septa or attenuated lung help differentiate it. (**d**) Axial CT image in lung window demonstrating pneumomediastinum, with curvilinear foci of gas located within the mediastinum adjacent to mediastinal structures (dashed black arrow pointing to right paratracheal gas). This can sometimes extend within the mediastinum near the pleura, mimicking a paramediastinal pneumothorax.

Accurate identification of pneumothorax is crucial, but several entities can mimic its appearance on imaging, leading to potential diagnostic errors. Radiologists should be aware of these common pitfalls on both chest radiography (CXR) and computed tomography (CT) (Figure 8 illustrates some of these common mimics and their differentiating features).

### 4.1. Chest Radiography Pitfalls

Skin Folds: These can create a pseudo-pleural line. They are differentiated by often extending beyond the thoracic cavity, appearing thicker or less sharp than a true pleural line, and having visible lung markings peripheral to them [26].

Bullous Disease/Large Cysts: Apical bullae are often misinterpreted as a loculated pneumothorax. A bulla is favored by a convex inner margin and the presence of faint lung markings or septations within the lucency [26].

Medical Equipment: Overlying lines from catheters, ECG leads, or patient gowns can create confusing linear opacities and require careful correlation with equipment positioning [26].

Artifacts: Motion or image processing can occasionally create spurious lines that mimic a pleural edge.

### 4.2. Computed Tomography Pitfalls

On CT, care must be taken to differentiate pneumothorax from the following mimics:

Large Bulla: Differentiating a large bulla from a loculated pneumothorax can be challenging. A bulla is favored by its intraparenchymal location (surrounded by attenuated lung), a convex inner margin, and the absence of a distinct visceral pleural line separate from the bulla wall. Multiplanar reformats (MPRs) are invaluable for this assessment [26].

Pneumomediastinum: Extensive pneumomediastinum can dissect along fascial planes adjacent to the pleura, mimicking a paramediastinal pneumothorax. CT will clearly show the gas to be confined within mediastinal planes rather than free in the pleural space.

Congenital Pulmonary Airway Malformations (CPAMs): In pediatric patients, large cystic CPAMs can be confused with pneumothorax. The presence of discernible cystic walls and internal septations helps differentiate a CPAM [27].

Peripheral Cavitary Lesions: A large, peripheral cavitating pneumonia or neoplasm can mimic a hydropneumothorax. A thicker, irregular wall and associated parenchymal abnormalities are clues to the correct diagnosis [26].

**Figure 9:**
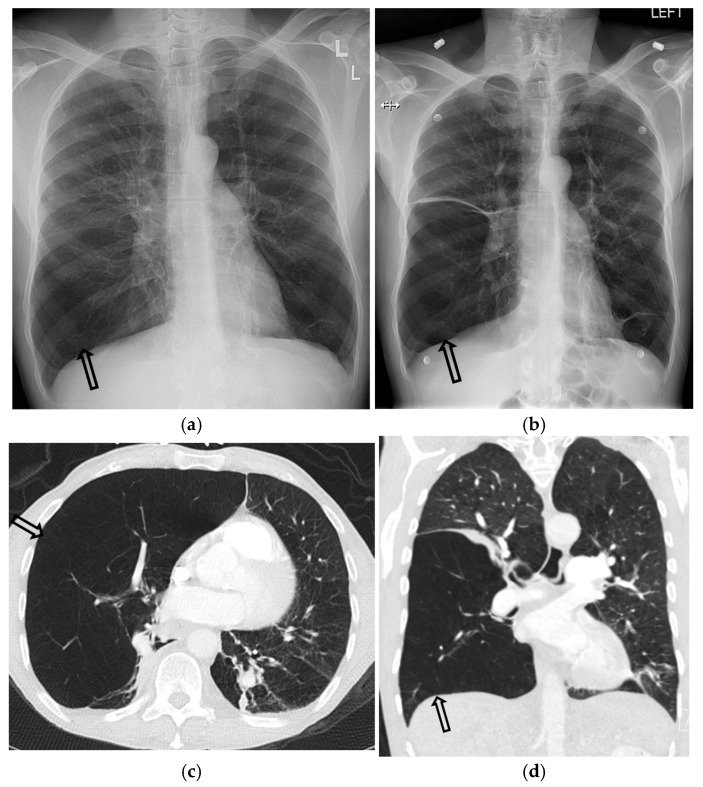
63-year-old male former smoker with a history of cocaine and intravenous ritalin use presents with chest pain. Initial frontal chest radiograph (**a**) and subsequent frontal chest radiograph performed 10 years later (**b**) show basilar-predominant hyperlucency of lungs (open black arrows). Axial and coronal CT images in lung window (**c**,**d**) show right-lower-lobe predominant panlobular emphysema (open black arrows), consistent with ritalin lung.

Panlobular Emphysema: Conditions like alpha-1 antitrypsin deficiency can cause diffuse hyperlucency that may obscure a small coexisting pneumothorax (Figure 9). Careful inspection for the visceral pleural line is key [28].

## 5. Underlying Etiologies of Secondary Spontaneous Pneumothorax (SSP): A Detailed Review

### 5.1. Infections

Pulmonary infections are a well-established cause of SSP, typically resulting from parenchymal necrosis, cyst formation, and subsequent visceral pleural rupture. Key infectious etiologies include tuberculosis, COVID-19, and opportunistic infections like *Pneumocystis jirovecii* pneumonia.

#### 5.1.1. Tuberculosis (TB)

**Figure 10:**
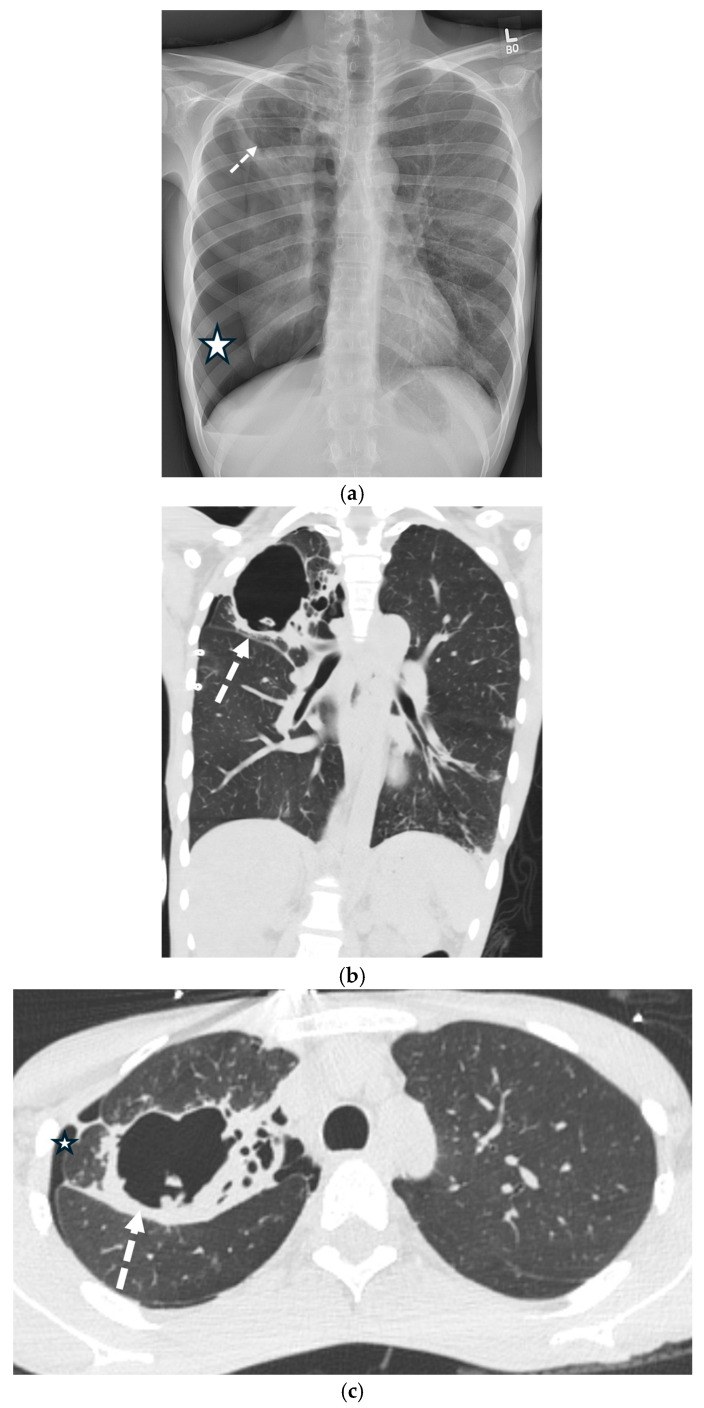
34 -year-old male presents with right chest pain. Frontal chest radiograph (**a**) demonstrates moderate-to-large right pneumothorax (large white star) and right-upper-lobe thick-walled cavitary lesion (white dashed arrow). Coronal and axial CT images in lung window (**b**,**c**) demonstrate small right pneumothorax (small star) status post-right chest tube placement. Note a large thick-walled cavitary lesion (white dashed arrow) in the right upper lobe with adjacent bronchiectasis. Sputum culture confirmed mycobacterium tuberculosis.

In endemic regions, tuberculosis is a leading cause of SSP, occurring in approximately 1% of patients with active disease, often in its advanced stages [21,29]. The mechanism involves the rupture of a tuberculous cavity or bleb into the pleural space (Figure 10). Common complications in these patients can include the formation of a bronchopleural fistula and empyema (Figure 10).

#### 5.1.2. COVID-19 Pneumonia

**Figure 11:**
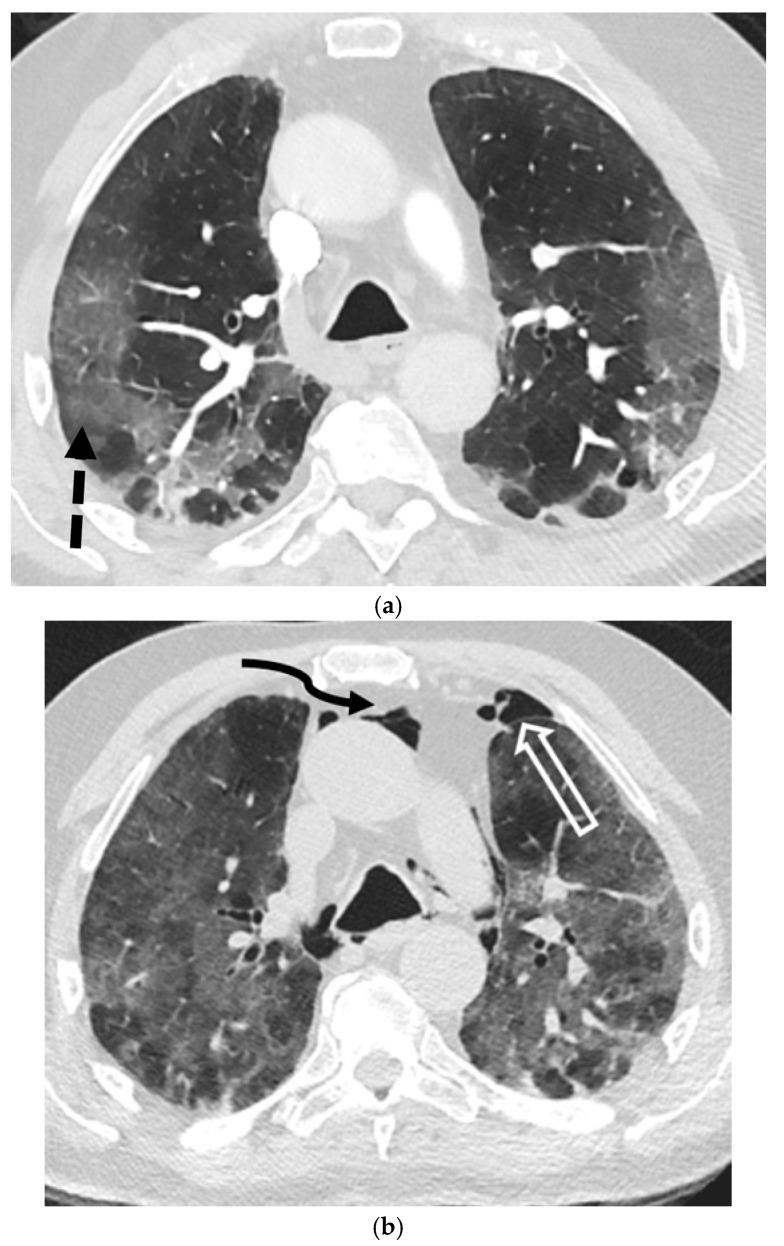

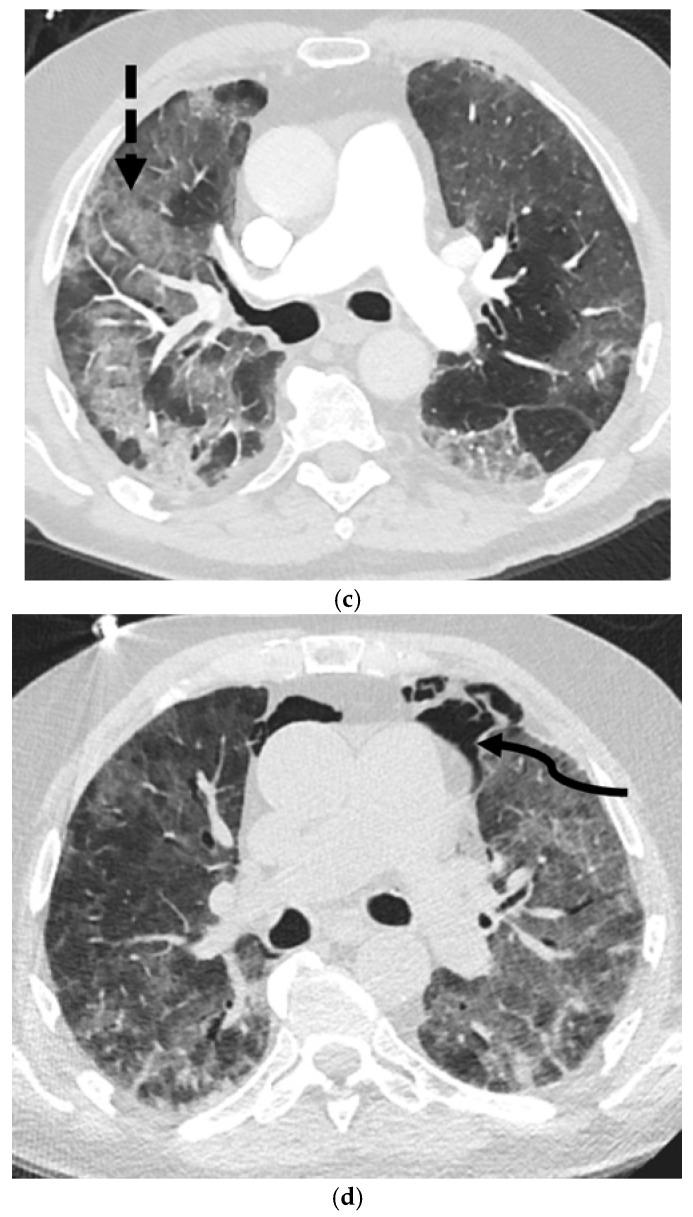
77-year-old male with a history of mantle cell lymphoma, recently hospitalized for COVID-19 pneumonia, now re-admitted 1 week later with persistent hypoxemic respiratory failure. Axial CT image in lung window at baseline (**a**) demonstrates peripheral predominant ground-glass opacities (dashed black arrow) in lungs consistent with known COVID-19 pneumonia. Repeat axial CT images in lung window obtained 1 week later (**b**–**d**) demonstrate worsening ground-glass opacities (dashed black arrow) in lungs along with a small new left pneumothorax (open white arrow) and pneumomediastinum (curved black arrow).

SP emerged as a notable complication during the COVID-19 pandemic, with a reported incidence of 1–1.4% in hospitalized patients. It is thought to result from diffuse alveolar damage, cystic lung changes, or barotrauma, particularly in patients requiring positive pressure ventilation [30]. The most common underlying CT findings of COVID-19 pneumonia are bilateral, peripheral ground-glass opacities (GGOs) or mixed consolidation and GGO (Figure 11). Nodular opacities with a peribronchovascular distribution are also seen [31].

#### 5.1.3. *Pneumocystis jirovecii* Pneumonia (PJP)

PJP, an opportunistic infection common in individuals with Acquired Immunodeficiency Syndrome (AIDS), carries a significant risk of SSP [32]. The primary mechanism is the rupture of thin-walled intrapulmonary cysts or pneumatoceles, a finding that can lead to pneumothorax in up to one-third of affected patients [21,32]. The classic imaging feature of PJP is diffuse, bilateral GGO, though consolidation, cysts, and nodules can also develop [33].

#### 5.1.4. Other Infections

**Figure 12:**
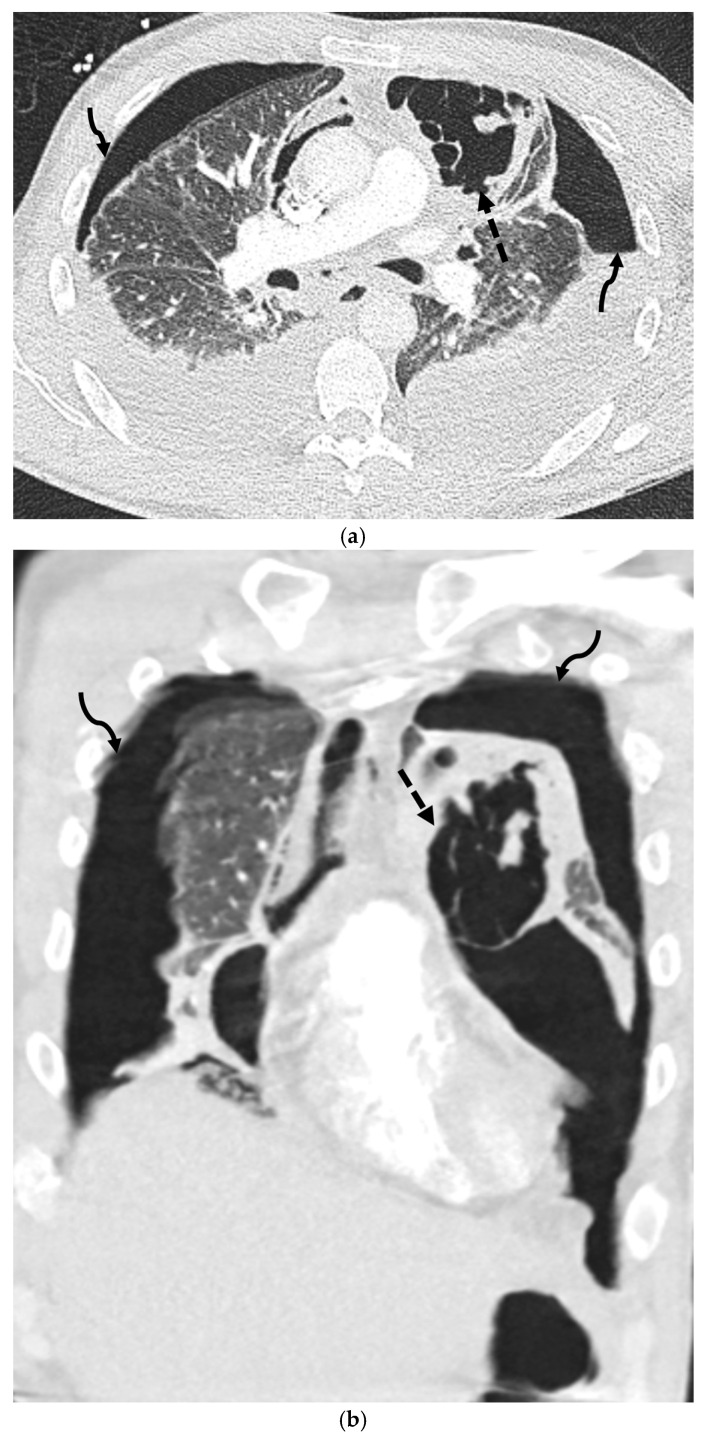
38-year-old male presenting with severe septic shock. Axial (**a**) and coronal (**b**) images from chest CT in lung windows demonstrates a cavitary lesion in the left upper lobe (dashed arrow) and bilateral hydropneumothoraces (curved arrows).

Necrotizing bacterial pneumonias caused by organisms such as *Staphylococcus aureus*, *Klebsiella pneumoniae*, and *Pseudomonas aeruginosa* can also lead to SSP through parenchymal destruction and fistula formation (Figure 12). Additionally, the rupture of pulmonary hydatid cysts is a rare infectious cause of pneumothorax [21].

### 5.2. Airway Diseases

#### 5.2.1. Chronic Obstructive Pulmonary Disease (COPD)

**Figure 13:**
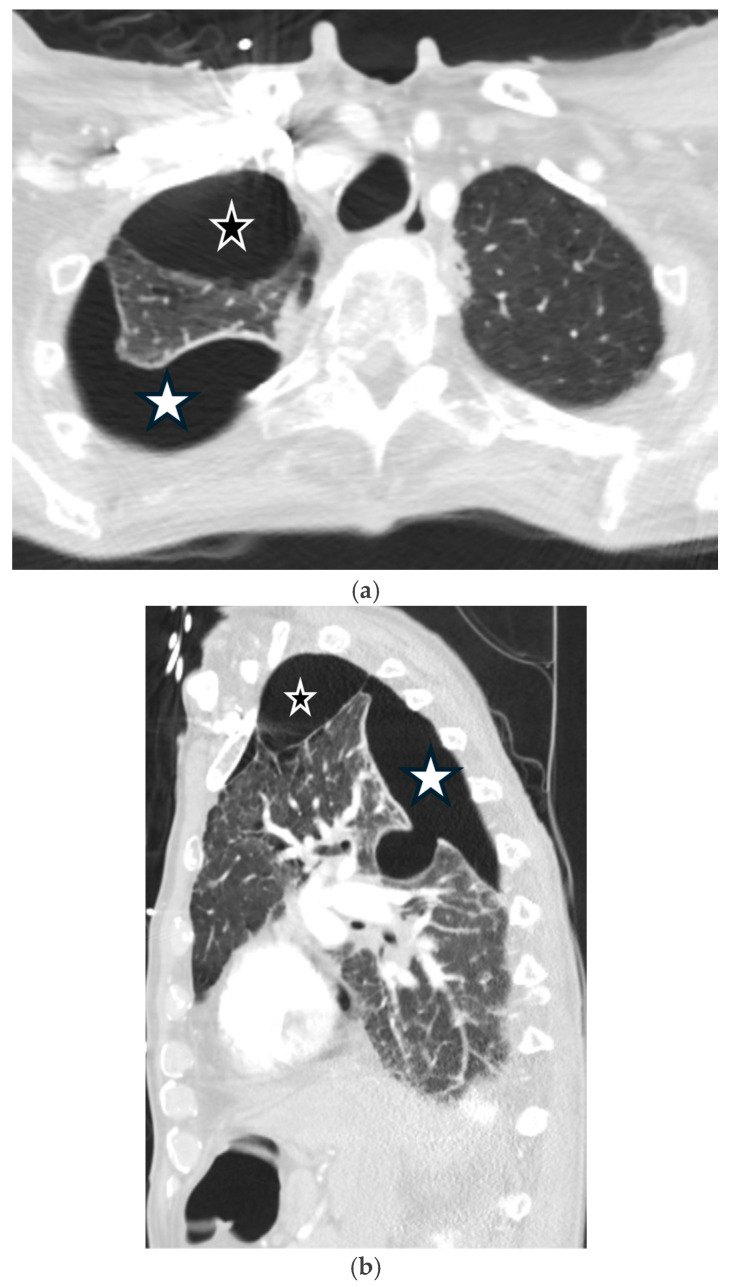

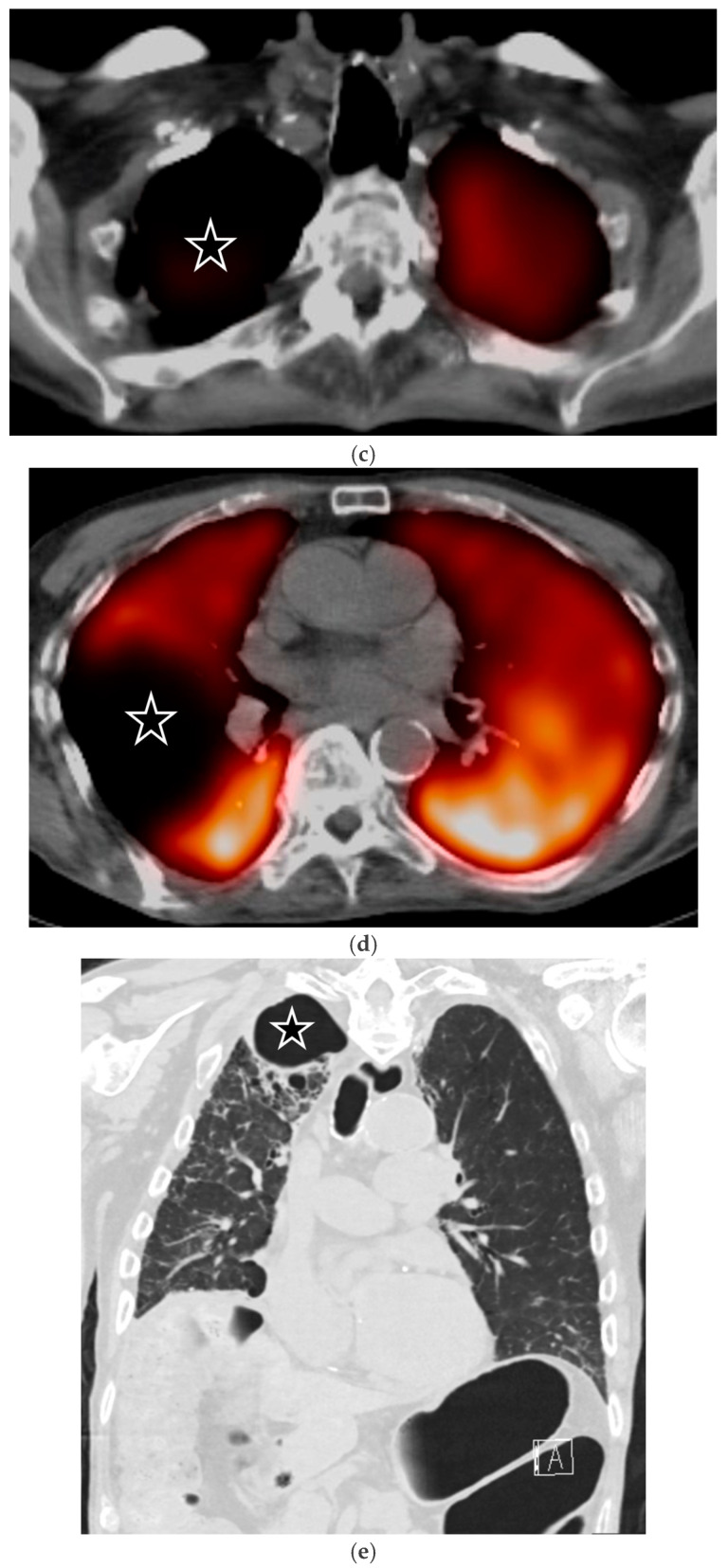
72-year-old male with bullous emphysema presents with dyspnea. Axial and sagittal CT images in lung window (**a**,**b**) demonstrate right apical bulla (black star) and loculated right pneumothorax (white star). Axial perfusion SPECT/CT images (**c**,**d**) reveal no radiotracer uptake in the right apical bleb or loculated right pneumothorax (black star). Subsequent coronal CT image in lung window (**e**) shows interval resolution of loculated pneumothorax after insertion of chest tube with persistence of right apical bulla (black star).

Chronic obstructive pulmonary disease (COPD) is the leading cause of SSP, resulting from the rupture of apical blebs or bullae. CT confirms the pneumothorax and demonstrates the characteristic underlying emphysema, bronchial wall thickening, and bullae [6,34] (Figure 13).

#### 5.2.2. Cystic Fibrosis (CF)

**Figure 14:**
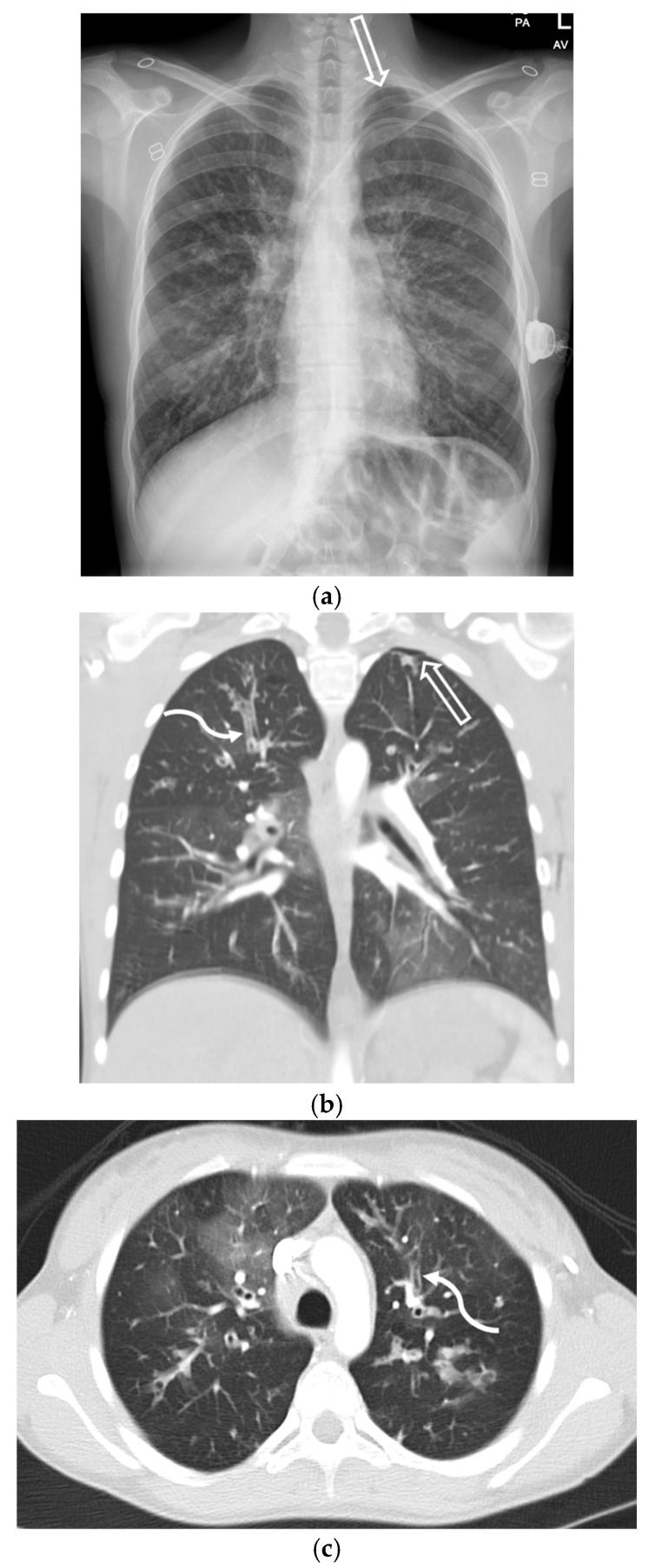
17-year-old male with cystic fibrosis presents with chest pain. Frontal chest radiograph (**a**) demonstrates small left pneumothorax (open white arrow) along with pulmonary hyperinflation and bilateral coarsened central linear interstitial markings. Coronal and axial CT images in lung window (**b**,**c**) show findings of cystic fibrosis including mosaic attenuation of lungs reflecting heterogeneous pulmonary air trapping, mild bronchiectatic changes predominantly affecting upper lobes, and marked bronchial wall thickening due to bronchitis (curved white arrow).

In cystic fibrosis (CF), SP has a high incidence and recurrence rate, caused by the rupture of apical subpleural cysts. Thoracic CT is characteristic, revealing the culprit cysts along with upper-lobe predominant bronchiectasis, bronchial wall thickening, and mucoid impaction [35,36,37] (Figure 14).

#### 5.2.3. Asthma

**Figure 15:**
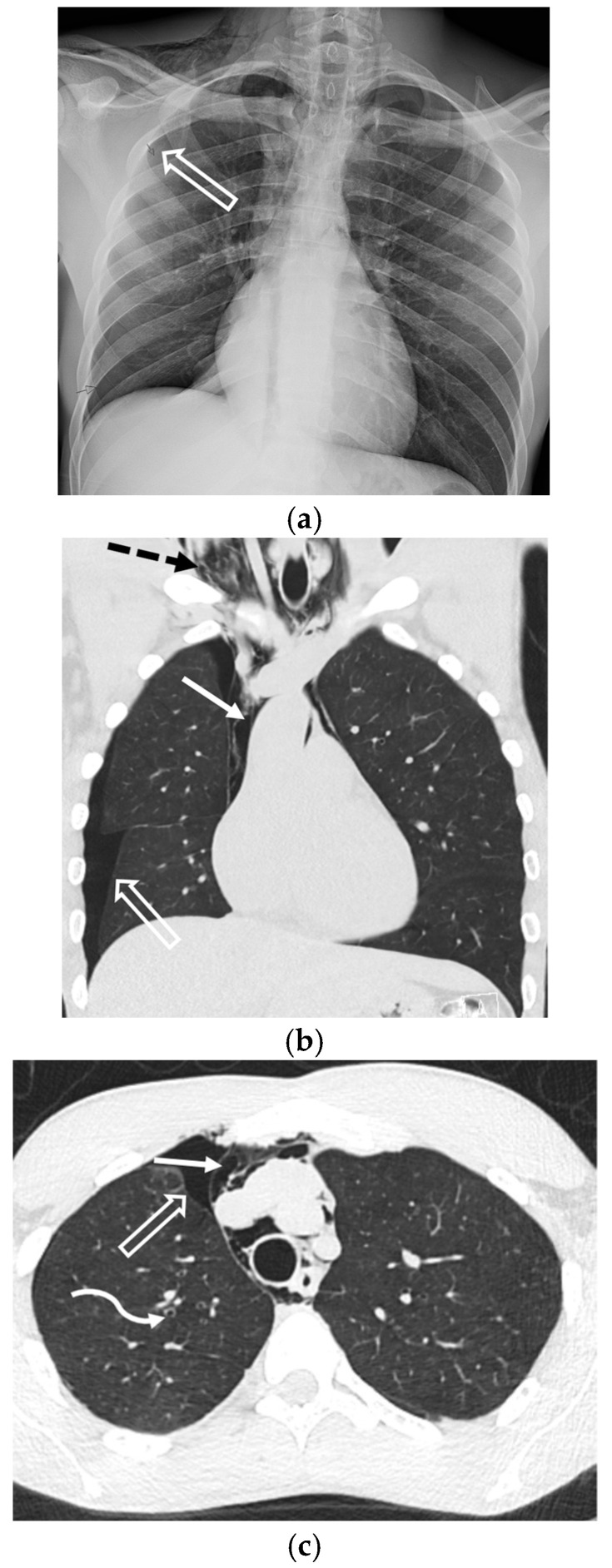
28-year-old male with asthma presents with chest pain. Frontal chest radiograph (**a**) demonstrates small-to-moderate right pneumothorax (open white arrow). There is also pneumomediastinum (solid white arrow) and subcutaneous emphysema (dashed black arrow), better evaluated on coronal and axial CT images in lung window (**b**,**c**). Axial CT image also shows diffuse bronchial wall thickening (curved white arrow) due to bronchitis.

SP is an infrequent complication of asthma, typically resulting from barotrauma. During a severe exacerbation, intense coughing or air trapping can lead to alveolar rupture. While chest imaging may be normal between attacks, CT performed during an acute episode often demonstrates the underlying bronchial wall thickening, mucous plugging, and air trapping that contribute to this risk [38,39] (Figure 15).

### 5.3. Malignancies

**Figure 16:**
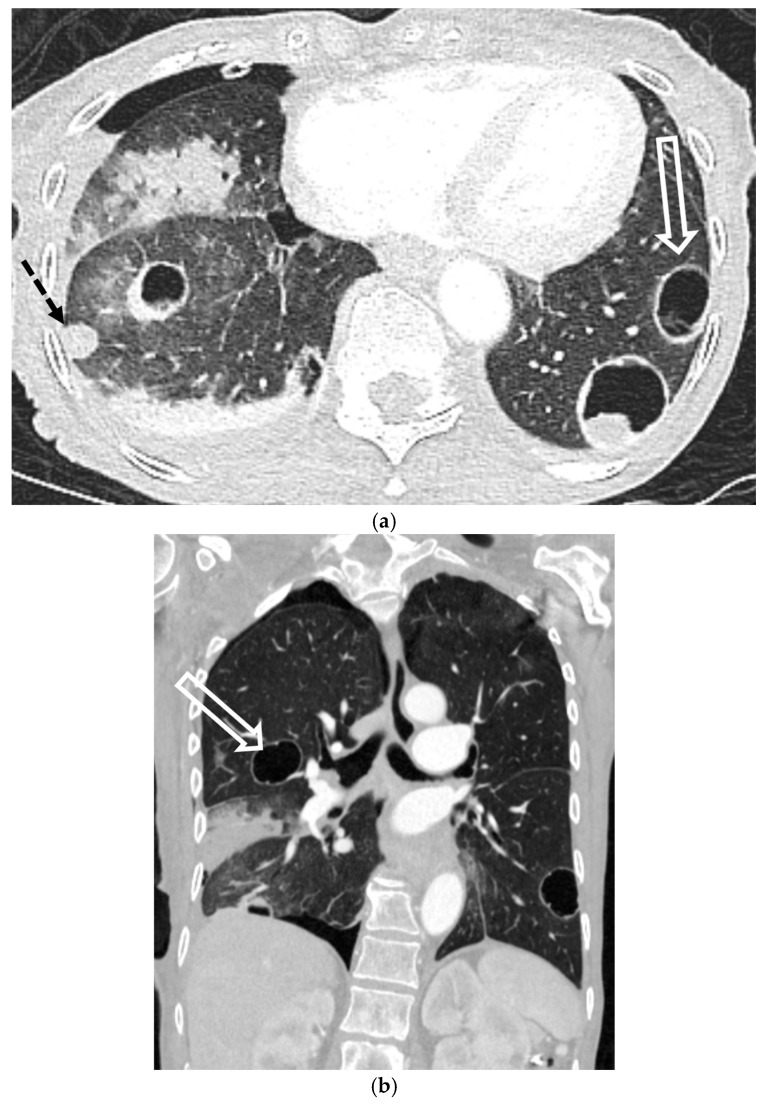
69-year-old female with a history of hepatocellular carcinoma presents with chest pain. Axial and coronal CT images in lung window (**a**,**b**) show small right pneumothorax, status post right chest tube placement, as well as multiple thick-walled cavitary lesions (open white arrows) and solid lung nodules.

**Figure 17:**
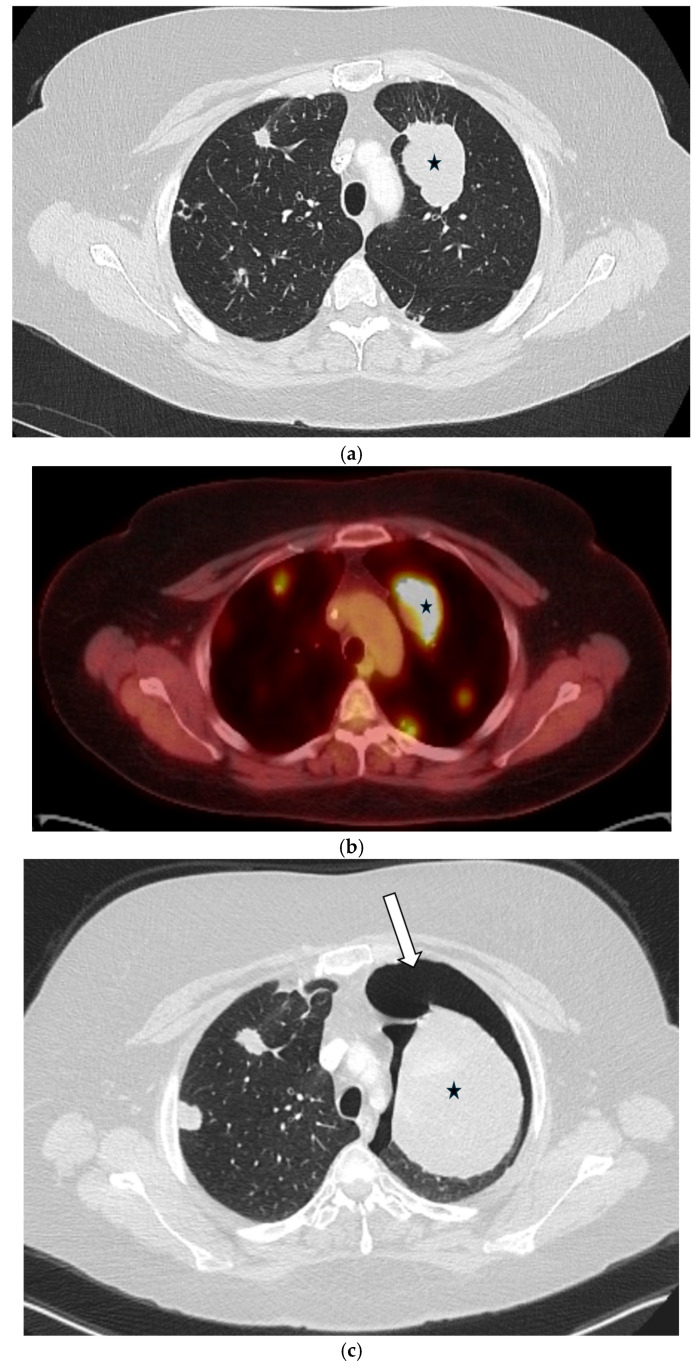
45-year-old female with a history of metastatic rhabdosarcoma. Baseline axial CT image in lung window (**a**) demonstrates multiple bilateral solid lung nodules and masses (black star) due to metastases. Baseline FDG-PET/CT image (**b**) shows avid radiotracer uptake (black star) in lung metastases. Subsequent axial CT image in lung window performed 2 months later (**c**) demonstrates interval worsening of metastatic disease (black star) with new small left pneumothorax (white arrow).

Malignancy accounts for up to 10% of SSP cases and may be the initial manifestation of an occult cancer [40,41]. The pneumothorax typically results from necrosis of a subpleural lesion, cavitation, or as a complication of therapy [40,42,43]. While most associated with sarcomas and primary lung cancer, cavitary metastases from other tumors can also be the cause [44,45,46]. CT is critical for identifying the responsible underlying nodules, masses, or cavitary lesions [42] (Figure 16 and Figure 17).

**Figure 18:**
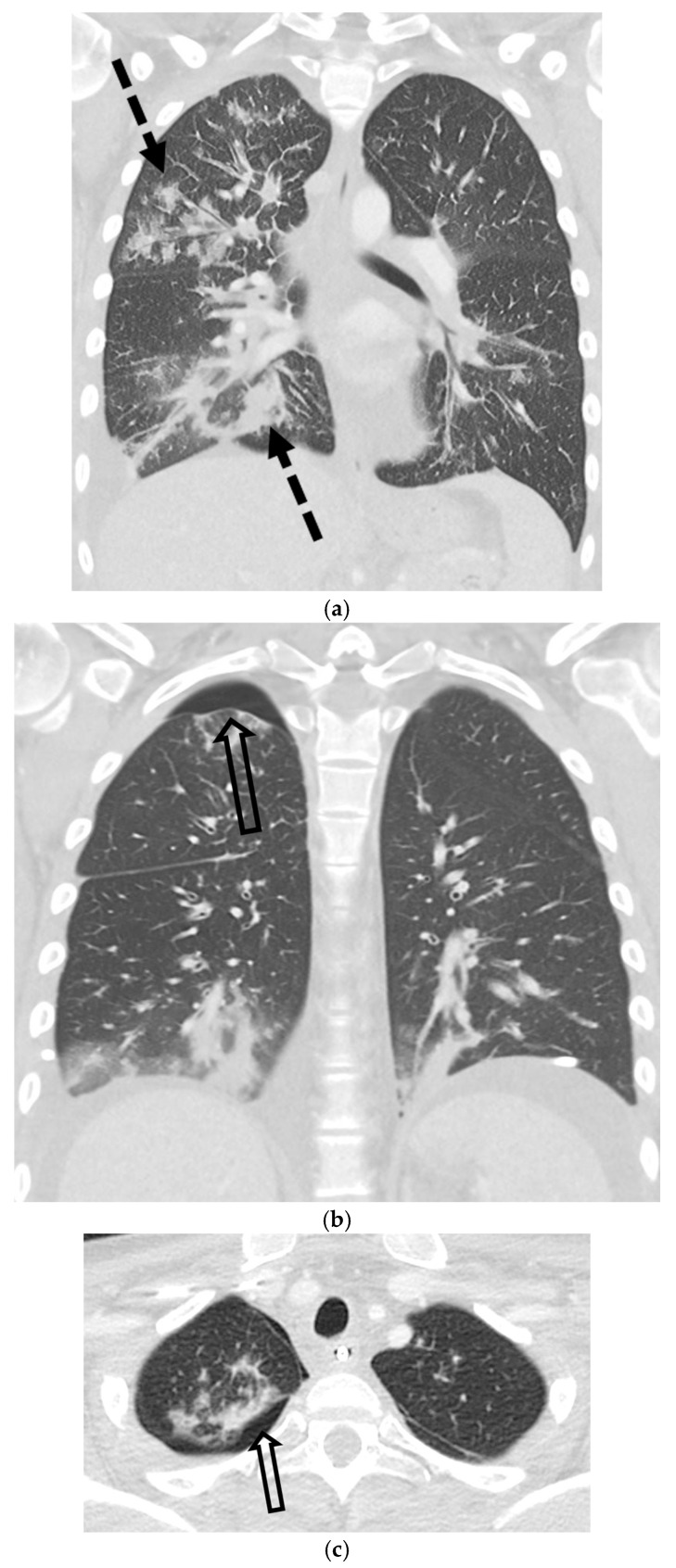

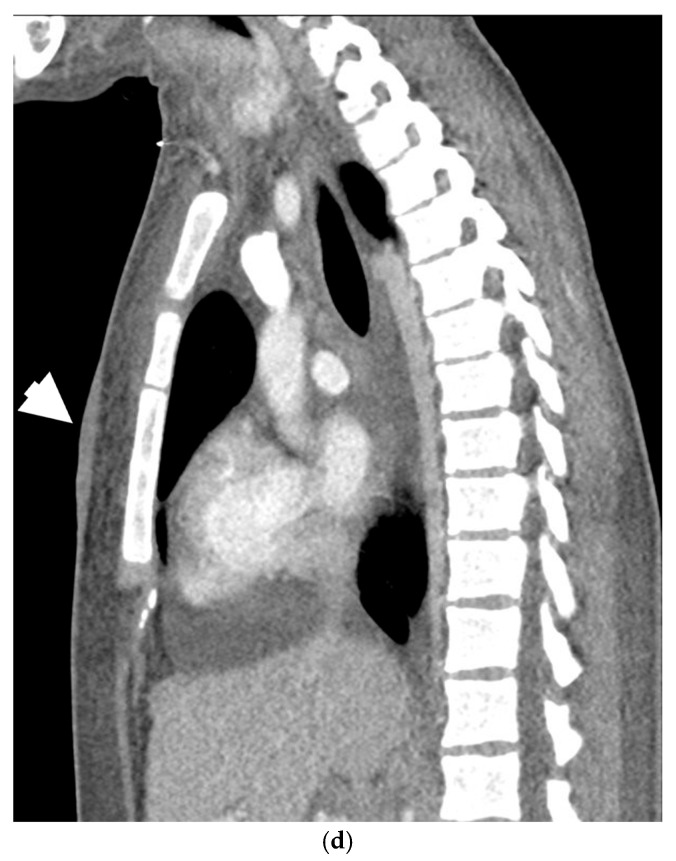
17-year-old male with a history of acquired immune deficiency syndrome and Kaposi’s sarcoma presents with respiratory distress. Coronal CT image in lung window (**a**) shows right-greater-than-left multifocal flame-shaped pulmonary opacities (dashed black arrows) consistent with pulmonary Kaposi’s sarcoma. Additional coronal and axial CT images in lung window (**b**,**c**) reveal a small right pneumothorax (open black arrow). Sagittal CT image in soft tissue window (**d**) demonstrates focal skin thickening in the anterior chest wall (white arrowhead), also in keeping with Kaposi’s sarcoma.

Kaposi sarcoma (KS), a Human Herpesvirus 8 (HHV-8) associated malignancy seen in patients with acquired immunodeficiency syndrome (AIDS), can also lead to SSP. While pulmonary KS has many imaging manifestations, pneumothorax is a less common but important complication. It is thought to arise from the necrosis of a subpleural KS nodule or from bulla formation secondary to parenchymal destruction by the tumor. CT will demonstrate the underlying ill-defined pulmonary nodules distributed along the bronchovascular bundles, in addition to the pneumothorax itself [47] (Figure 18).

### 5.4. Interstitial Lung Diseases (ILDs)

Interstitial lung diseases (ILDs) encompass a diverse group of disorders characterized by inflammation and fibrosis of the lung parenchyma. Spontaneous pneumothorax is a well-recognized complication, often portending a poor prognosis. While some forms are idiopathic (e.g., IPF) or associated with systemic granulomatous disease (e.g., sarcoidosis), ILD is also a frequent and significant manifestation of several connective tissue diseases.

#### 5.4.1. Idiopathic Pulmonary Fibrosis (IPF)

**Figure 19:**
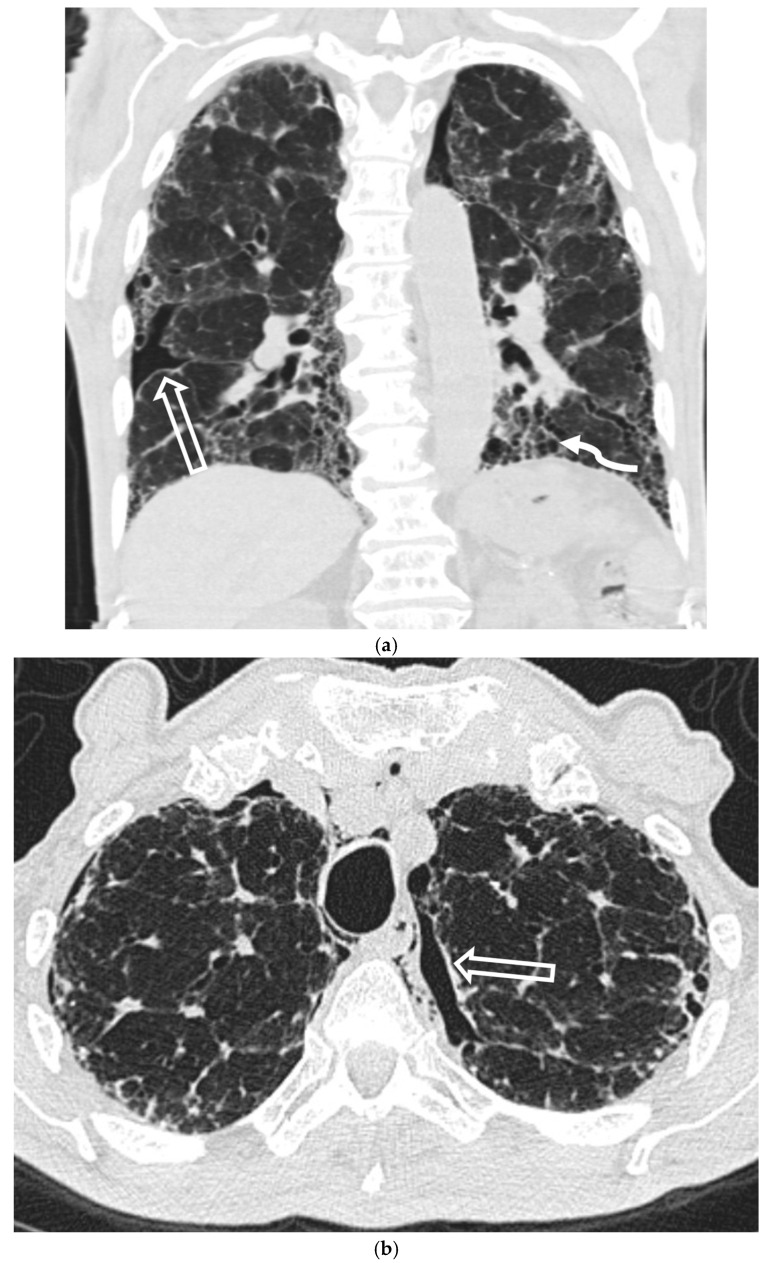
84-year-old male with a history of interstitial lung disease presents with dyspnea. Coronal and axial CT images in lung window (**a**,**b**) show peripheral basilar-predominant reticulation (curved white arrow), mild ground-glass opacity, architectural distortion, traction bronchiectasis, and honeycombing characteristic of idiopathic pulmonary fibrosis, along with small bilateral pneumothoraces (open white arrows) and small pneumomediastinum.

Idiopathic pulmonary fibrosis (IPF) is the second-most-common cause of SSP, occurring in 2–20% of patients and portending a poor prognosis [48]. The pneumothorax is typically caused by the rupture of subpleural honeycombed cysts or fibrotic blebs. CT is essential for diagnosis, revealing the characteristic usual interstitial pneumonia (UIP) pattern of peripheral and basilar-predominant interstitial fibrosis, traction bronchiectasis, and honeycombing that underlies the pneumothorax (Figure 19).

#### 5.4.2. Lymphoid Interstitial Pneumonia (LIP)

SP is a reported, albeit uncommon, complication of lymphoid interstitial pneumonia (LIP), an interstitial lung disease associated with autoimmune disorders such as Sjogren’s syndrome and AIDS. The pneumothorax is thought to result from the rupture of the thin-walled perivascular cysts that are a key feature of the disease. CT typically demonstrates these cysts, which are often accompanied by ground-glass opacities and centrilobular nodules, predominantly in the lower lung zones [49,50,51].

#### 5.4.3. Sarcoidosis

**Figure 20:**
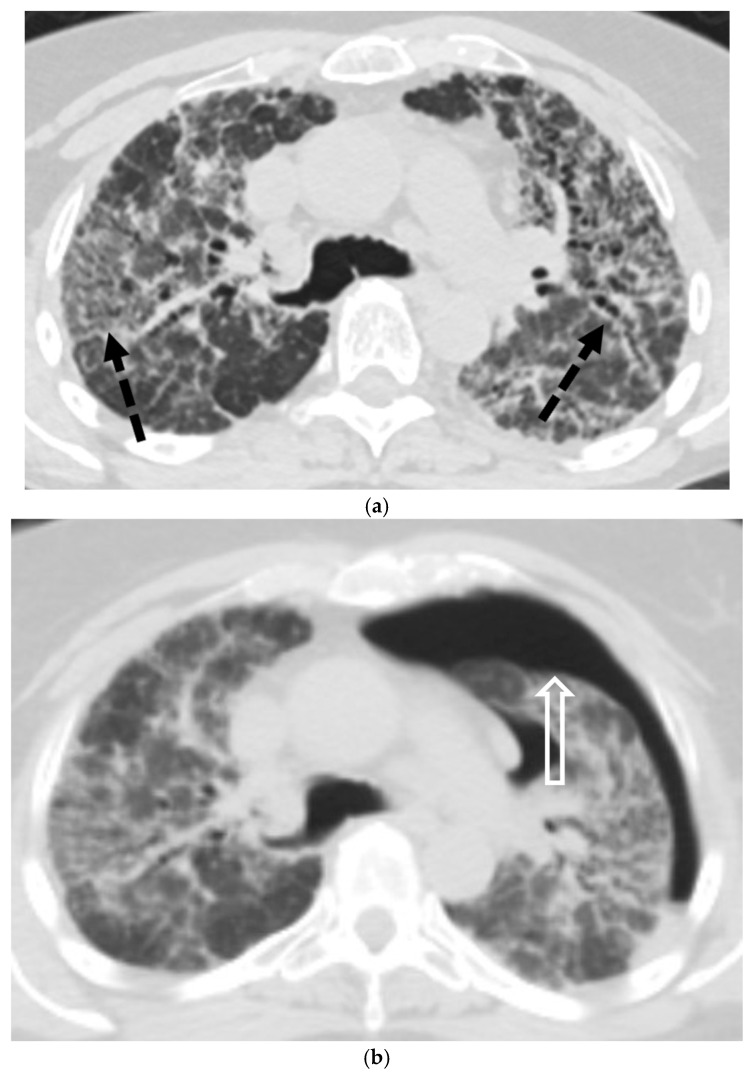

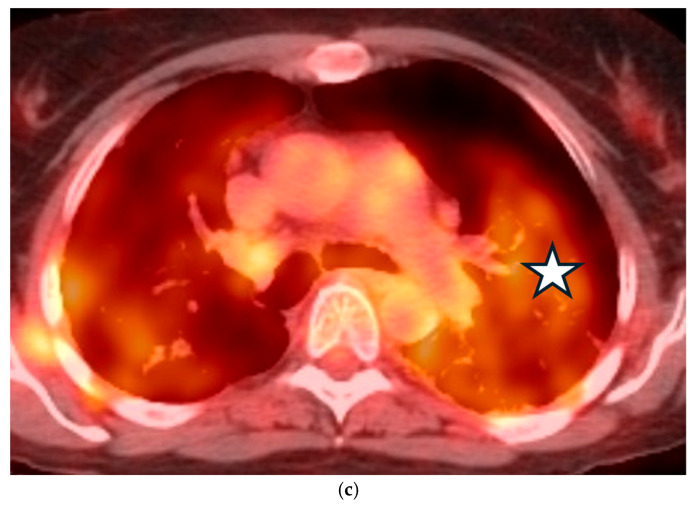
65-year-old female with advanced pulmonary sarcoidosis undergoes pre-surgical evaluation before lung transplantation. Axial CT image in lung window at baseline (**a**) demonstrates widespread upper-lung-zone predominant fibrotic interstitial lung disease as manifested by areas of reticulation, ground-glass opacity, architectural distortion, and traction bronchiectasis (dashed black arrows). Axial CT image in lung window from cardiac FDG-PET/CT performed 1 year later (**b**) shows a new moderate left pneumothorax (open white arrow). A fused PET/CT image from the same cardiac FDG-PET/CT (**c**) demonstrates inflammatory FDG uptake in the lungs (white star) as well as FDG-avid mediastinal and bilateral hilar lymph nodes.

Sarcoidosis is a rare cause of SP (~2% of patients) but is an important consideration in young non-smokers. The mechanism is typically rupture of a subpleural bleb or necrosis of a granuloma, usually in late-stage fibrotic disease [52]. CT reveals the underlying cause by demonstrating the characteristic upper- and mid-lung zone predominant fibrosis, architectural distortion, and honeycombing [53] (Figure 20).

### 5.5. Diffuse Cystic Lung Diseases

The diffuse cystic lung diseases are significant causes of secondary spontaneous pneumothorax (SSP). In these conditions, the common underlying mechanism is the rupture of the characteristic thin-walled cysts, leading to air leakage into the pleural space

#### 5.5.1. Pulmonary Langerhans Cell Histiocytosis (PLCH)

**Figure 21:**
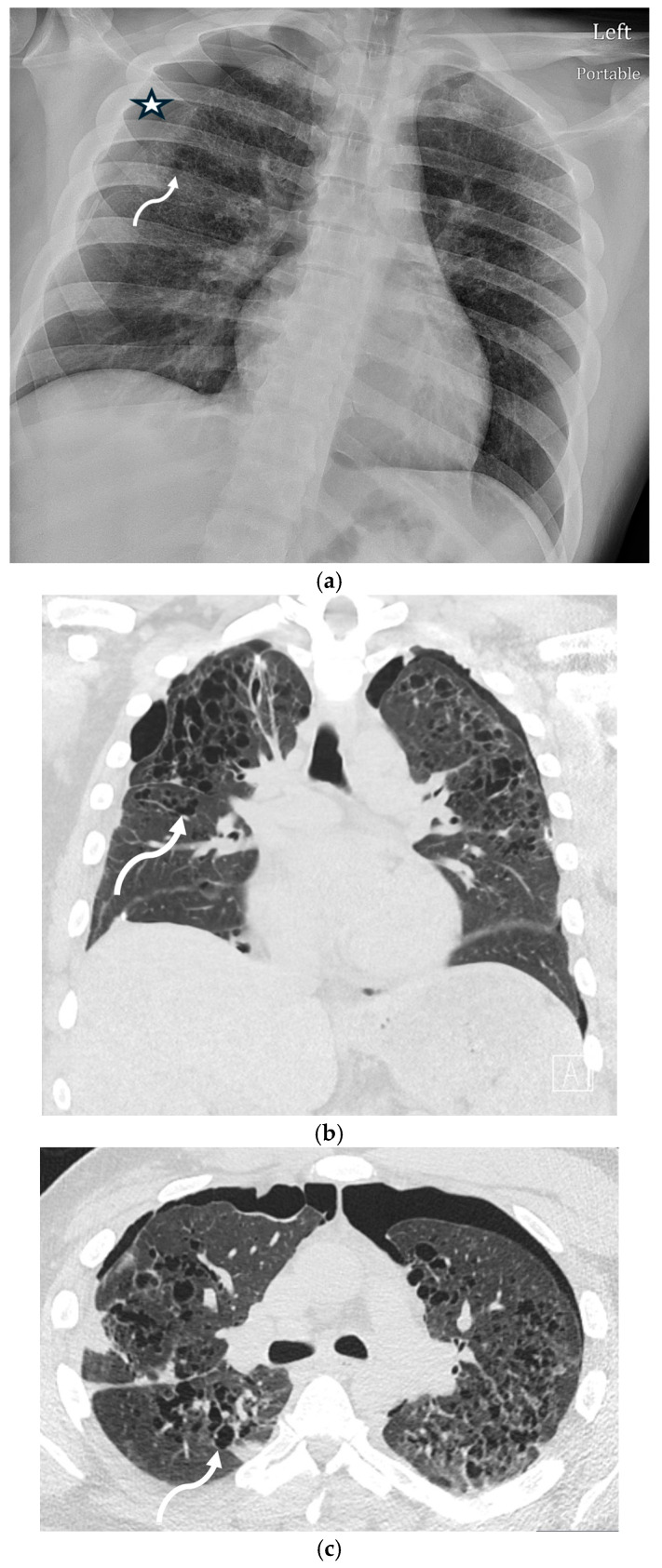
Pulmonary Langerhans cell histiocytosis in a 26-year-old male. (**a**) Frontal radiograph shows a small right pneumothorax (star) and upper-lobe interstitial and cystic changes (curved arrow). (**b**,**c**) Coronal and axial CT images confirm upper-lobe predominant, bizarre-shaped cysts (curved arrows) with associated interstitial thickening, moderate left pneumothorax, and small loculated right hydropneumothorax.

PLCH is a rare, smoking-related disease where SP is a common presenting feature, particularly in young adults [54,55]. Since pneumothorax can be the initial manifestation of the disease, CT evaluation is crucial. The characteristic CT findings are a combination of nodules and bizarrely shaped cysts, with a distinct predominance for the upper and mid-lung zones and relative sparing of the lung bases [54,55,56] (Figure 21).

#### 5.5.2. Lymphangioleiomyomatosis (LAM)

**Figure 22:**
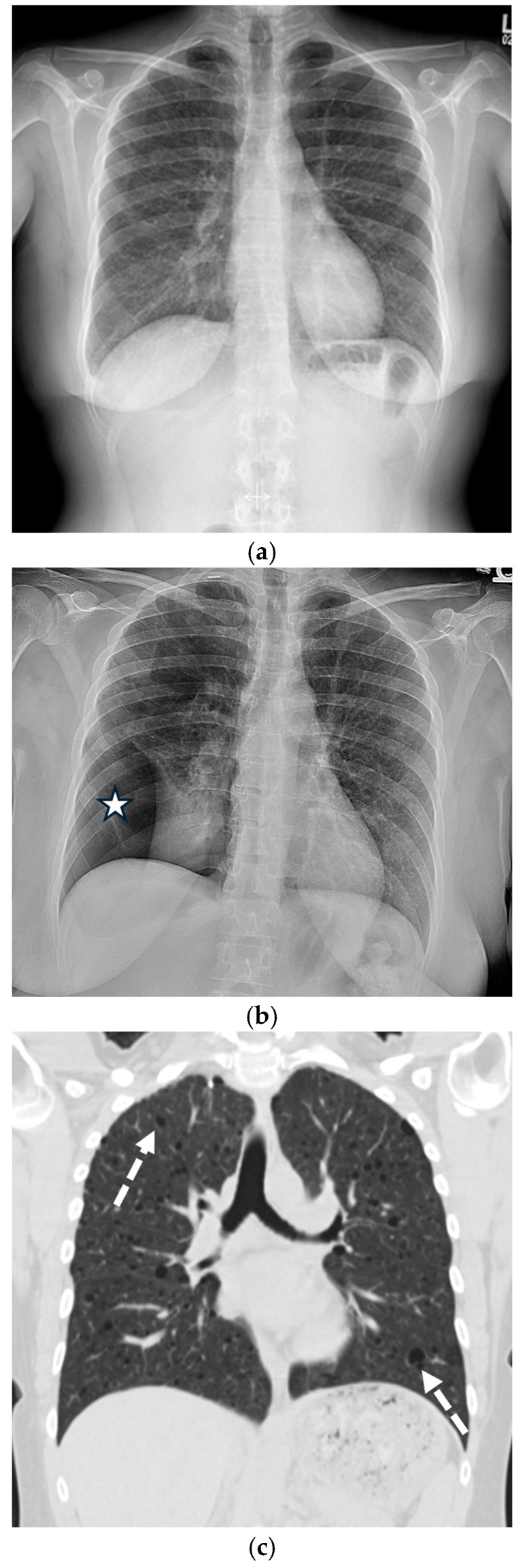
44-year-old female with LAM presents with chest pain. A frontal chest radiograph from 2 years ago (**a**) shows mild pulmonary interstitial prominence. Subsequent frontal chest radiograph (**b**) reveals a new moderate right pneumothorax (white star) with partial right-lung atelectasis. Coronal CT image in lung window (**c**) demonstrates multiple diffusely distributed thin-walled cysts (dashed white arrows) with normal areas of intervening lung.

Spontaneous pneumothorax (SP) is a hallmark of lymphangioleiomyomatosis (LAM), a cystic lung disease that occurs almost exclusively in women of reproductive age, either sporadically or in association with tuberous sclerosis complex (TSC). CT is diagnostic, revealing numerous thin-walled, round cysts distributed diffusely throughout the lungs, surrounded by otherwise normal-appearing lung parenchyma [57,58,59] (Figure 22).

#### 5.5.3. Birt–Hogg–Dubé Syndrome (BHDS)

**Figure 23:**
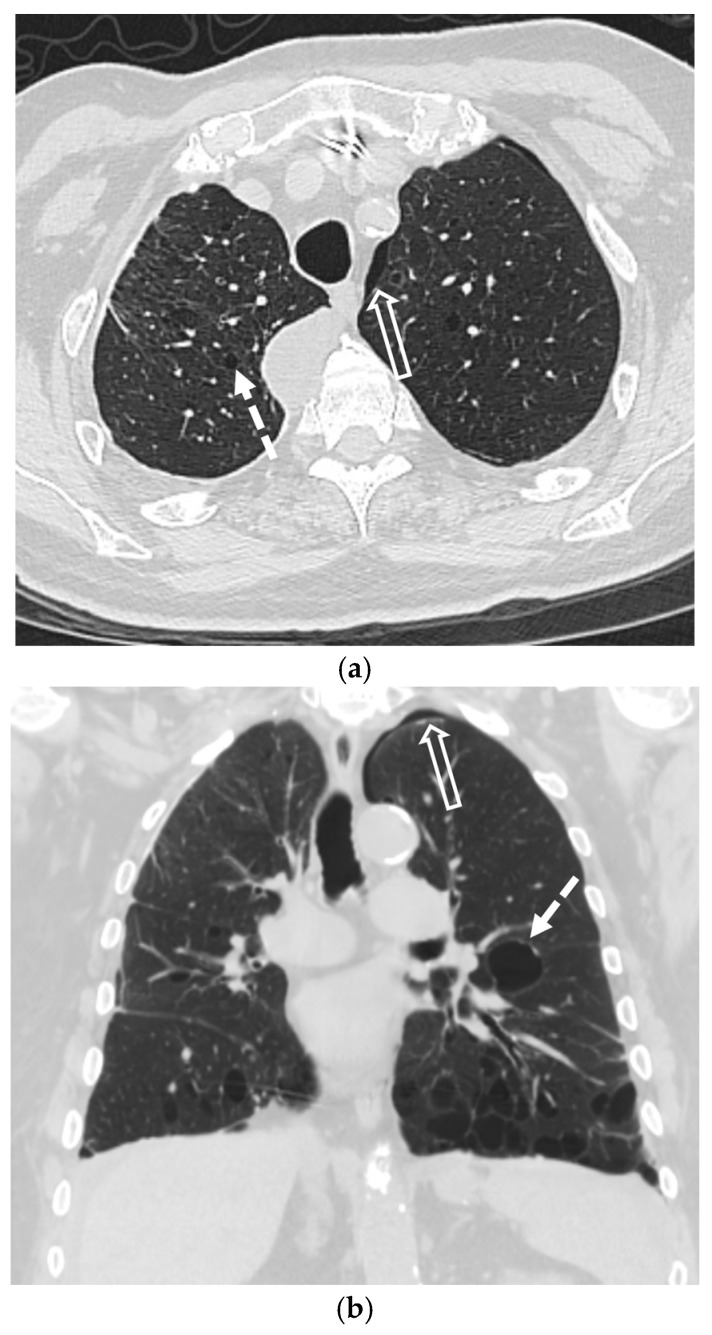
81-year-old male with Birt–Hogg–Dubé syndrome presents with left-sided chest pain. Axial and coronal CT images in lung window (**a**,**b**) demonstrate multiple bilateral thin-walled pulmonary cysts (dashed white arrows) of varying size with basilar predominance, along with trace left apical pneumothorax (open white arrow).

Birt–Hogg–Dubé syndrome (BHDS) is an autosomal dominant disorder characterized by a triad of lung cysts, renal neoplasms, and skin lesions [60]. SP is a major feature of the syndrome, affecting 22–41% of individuals due to the rupture of the characteristic thin-walled pulmonary cysts [61,62]. On CT, these cysts are a key diagnostic clue, typically appearing as elliptical or lentiform shapes with a notable basilar and subpleural distribution [17,63] (Figure 23).

### 5.6. Connective Tissue Diseases

#### 5.6.1. Rheumatoid Arthritis

**Figure 24:**
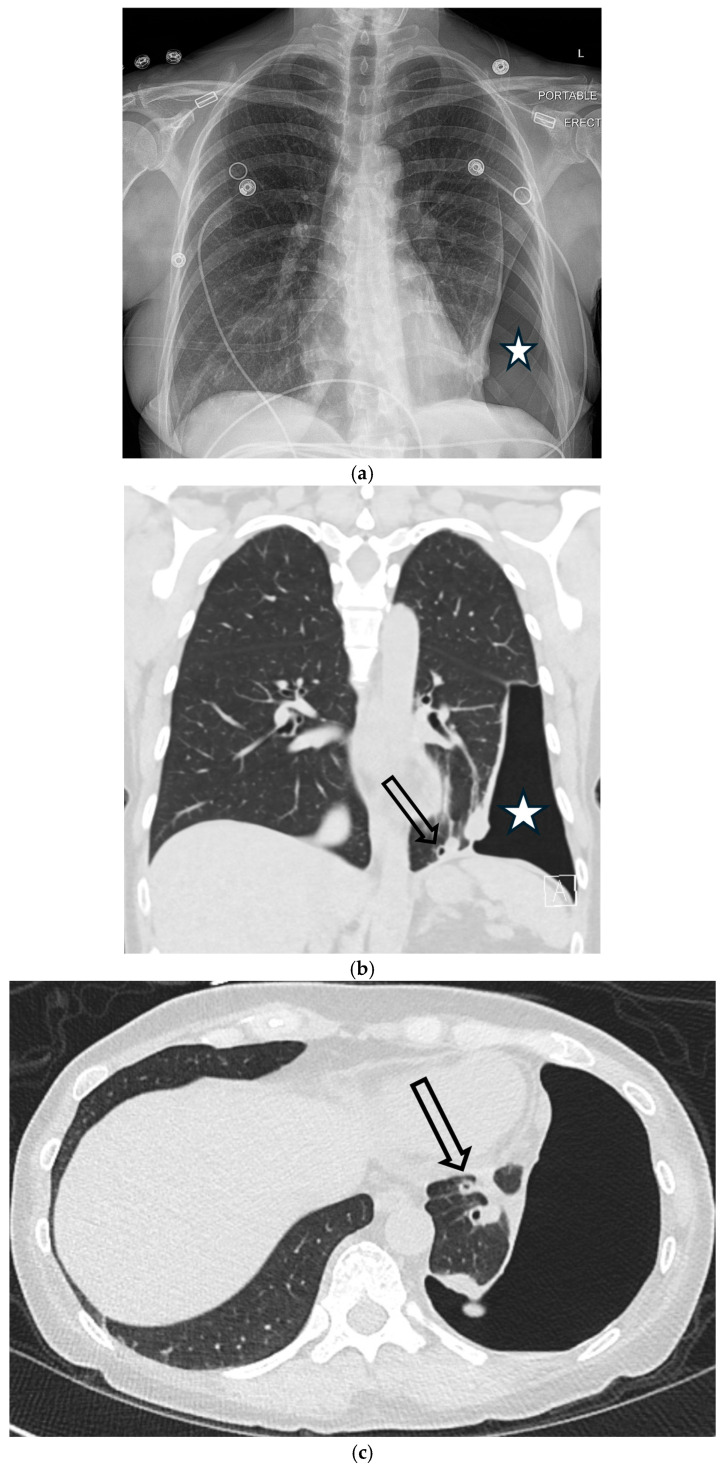
45-year-old female with rheumatoid arthritis presents with acute cough and chest pain. Frontal chest radiograph (**a**) demonstrates a large left basilar pneumothorax (white star) with partial atelectasis of the left lung. Coronal and axial CT images in lung window (**b**,**c**) again show left pneumothorax (white star), along with subpleural predominant solid and partially cavitary pulmonary nodules (open black arrow), in keeping with necrobiotic rheumatoid nodules.

SP in rheumatoid arthritis (RA) results from ruptured necrobiotic nodules or blebs in the setting of RA-ILD. CT identifies the pneumothorax and underlying cavitary nodules or fibrotic changes [64,65,66] (Figure 24).

#### 5.6.2. Systemic Sclerosis

**Figure 25:**
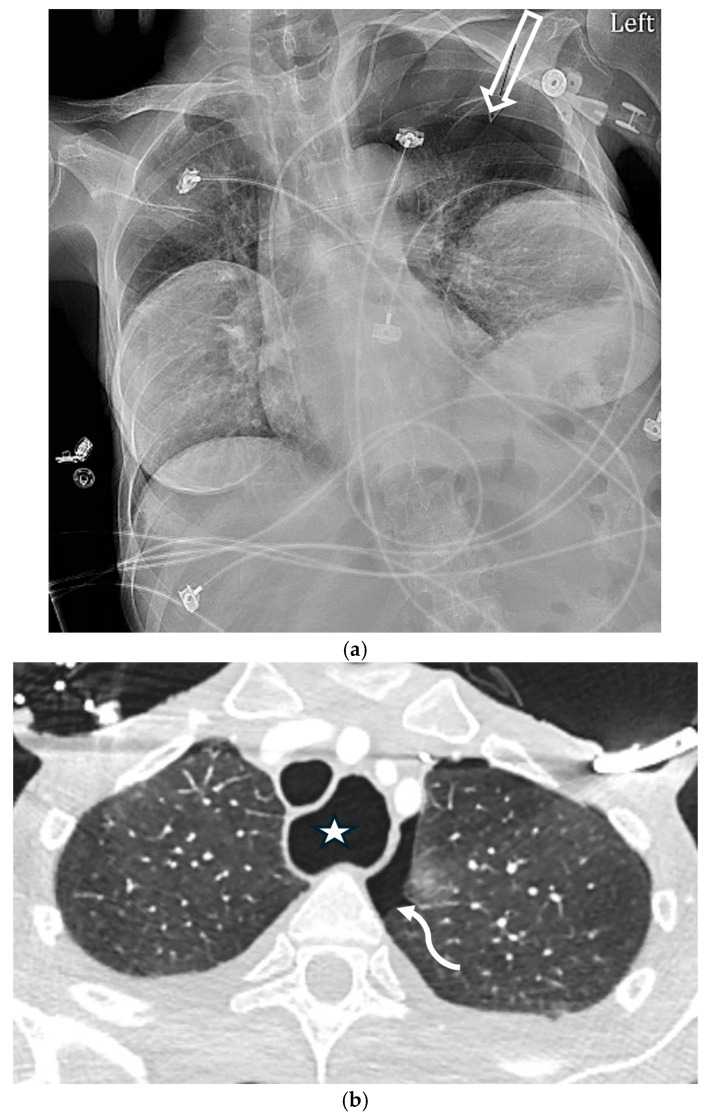

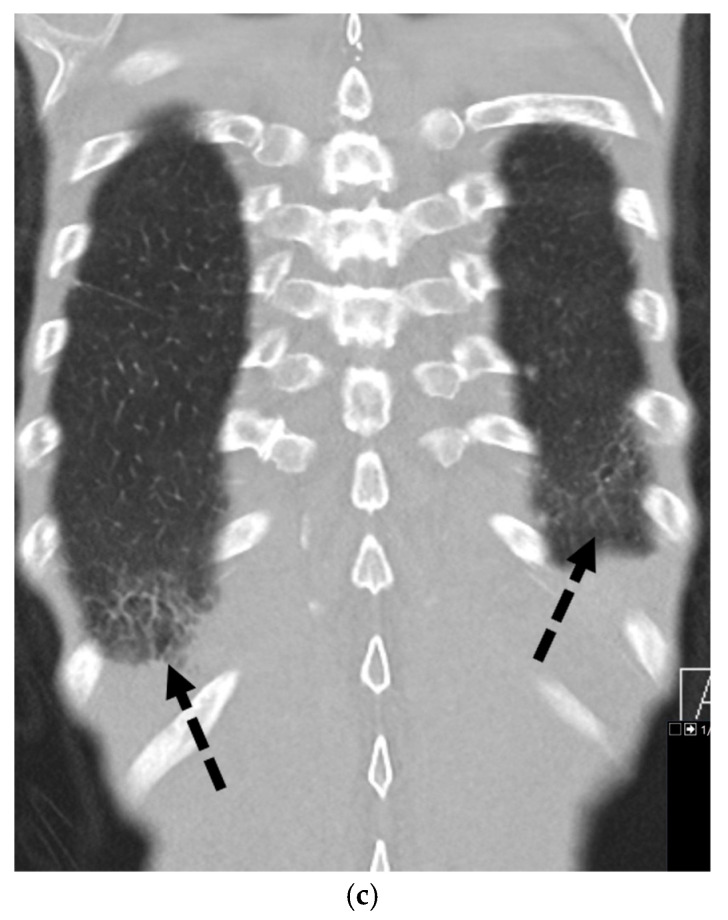
61-year-old female with systemic sclerosis. Frontal chest radiograph (**a**) shows a small left apical pneumothorax (open white arrow). Axial CT image in lung window (**b**) demonstrates diffusely dilated esophagus (white star) and small left pneumothorax (curved white arrow). Coronal CT image in lung window (**c**) shows basilar predominant peripheral reticulation with mild ground-glass opacity and bronchiolectasis without honeycombing (dashed black arrows), likely reflecting non-specific interstitial pneumonia (NSIP) in the setting of systemic sclerosis.

A rare complication of systemic sclerosis-associated ILD, SP is caused by the rupture of bullae or honeycombed cysts. Key CT findings include the underlying fibrotic ILD and an often strikingly dilated esophagus [67,68] (Figure 25).

#### 5.6.3. Ankylosing Spondylitis

SP is a rare manifestation, resulting from the formation and rupture of apical bullae in the context of upper-lobe fibrosis [69,70].

#### 5.6.4. Marfan’s Syndrome

**Figure 26:**
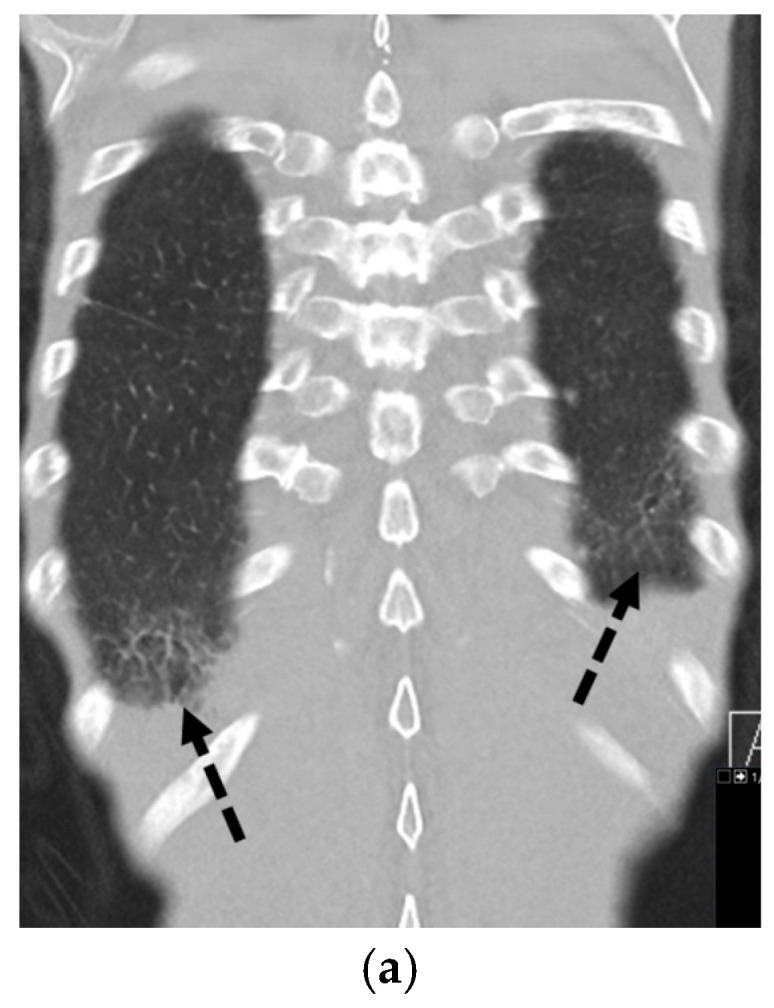

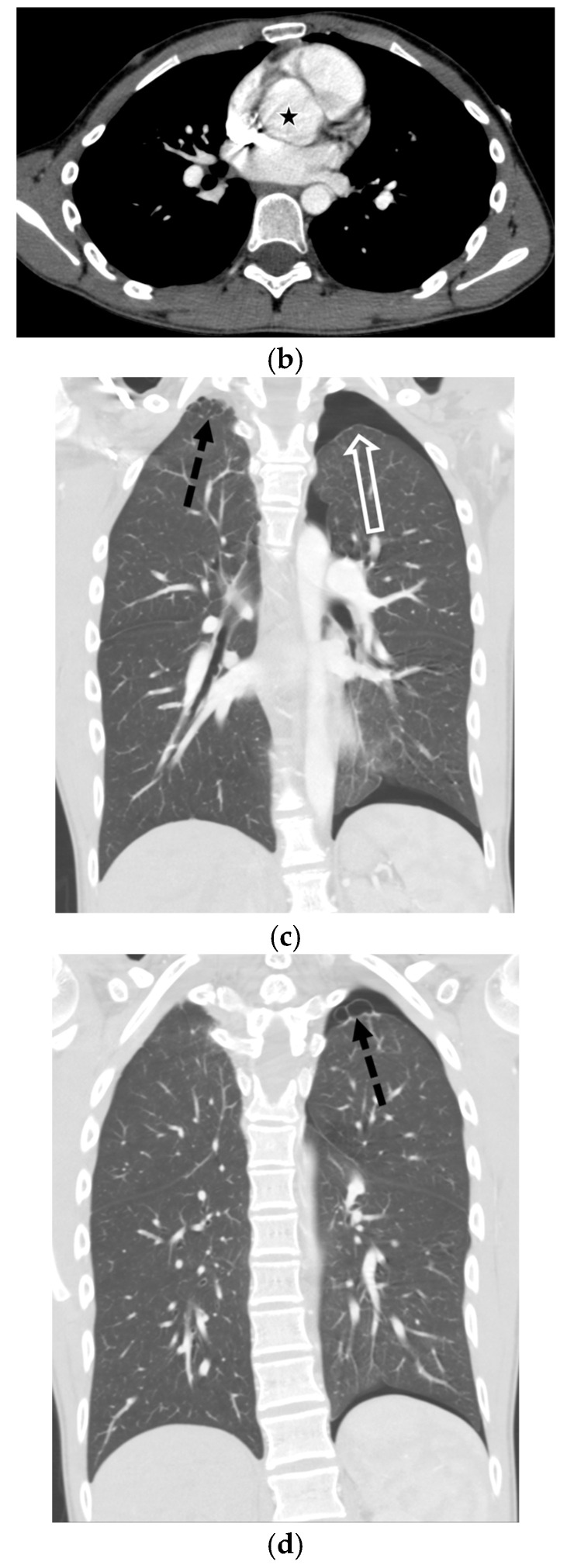
16-year-old male with Marfan’s syndrome presents with left-sided chest pain. Frontal chest radiograph (**a**) demonstrates moderate left pneumothorax (open black arrow) along with increased craniocaudal-to-transverse ratio of the thorax. Axial contrast-enhanced CT image in soft tissue window (**b**) shows dilated aortic root (black star). Coronal CT images in lung window (**c**,**d**) reveal biapical blebs (dashed black arrow) and moderate left pneumothorax (open white arrow).

SP is a key diagnostic feature of Marfan’s syndrome, caused by the rupture of apical bullae from an inherent collagen defect. Radiologists may suggest the diagnosis when a pneumothorax is seen with a dilated aortic root, scoliosis, or pectus deformity [17,71,72,73] (Figure 26).

#### 5.6.5. Ehlers–Danlos Syndrome (EDS)

**Figure 27:**
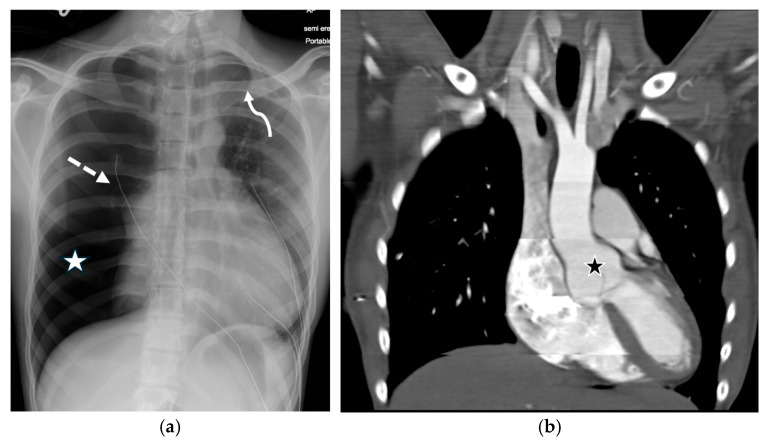
17-year-old male with Ehlers–Danlos syndrome presents with chest pain. Frontal chest radiograph (**a**) shows large right-sided pneumothorax (white star) under tension (as manifested by leftward mediastinal shift and inferior diaphragmatic shift), small left-sided pneumothorax (curved white arrow), and right apical bleb (dashed white arrow). Coronal contrast-enhanced CT image in soft tissue window (**b**) demonstrates dilated aortic root (black star).

In vascular EDS (vEDS), poor tissue integrity leads to a high prevalence of SP, often with concomitant hemothorax. Associated life-threatening arterial aneurysms or dissections may also be evident on chest imaging [17,74] (Figure 27).

### 5.7. Miscellaneous Causes

#### 5.7.1. Endometriosis

**Figure 28:**
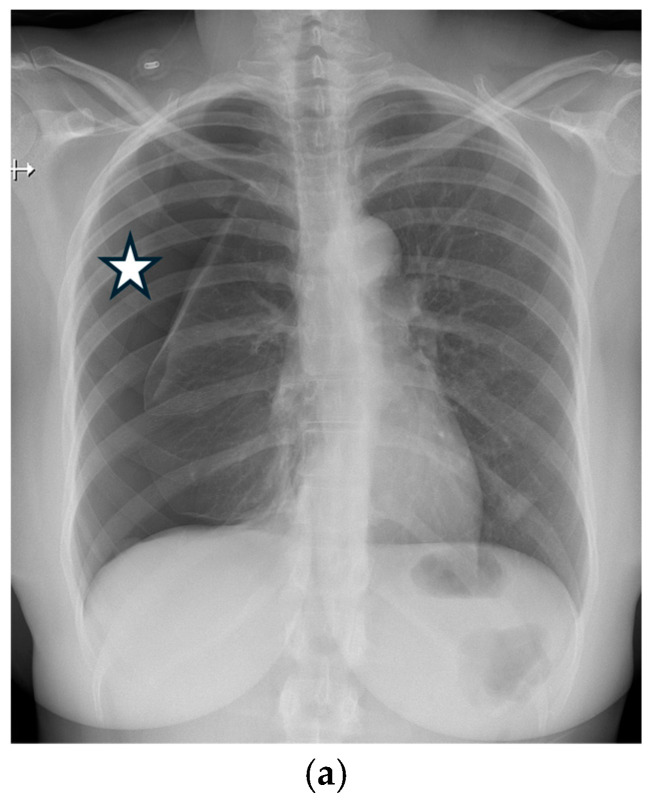

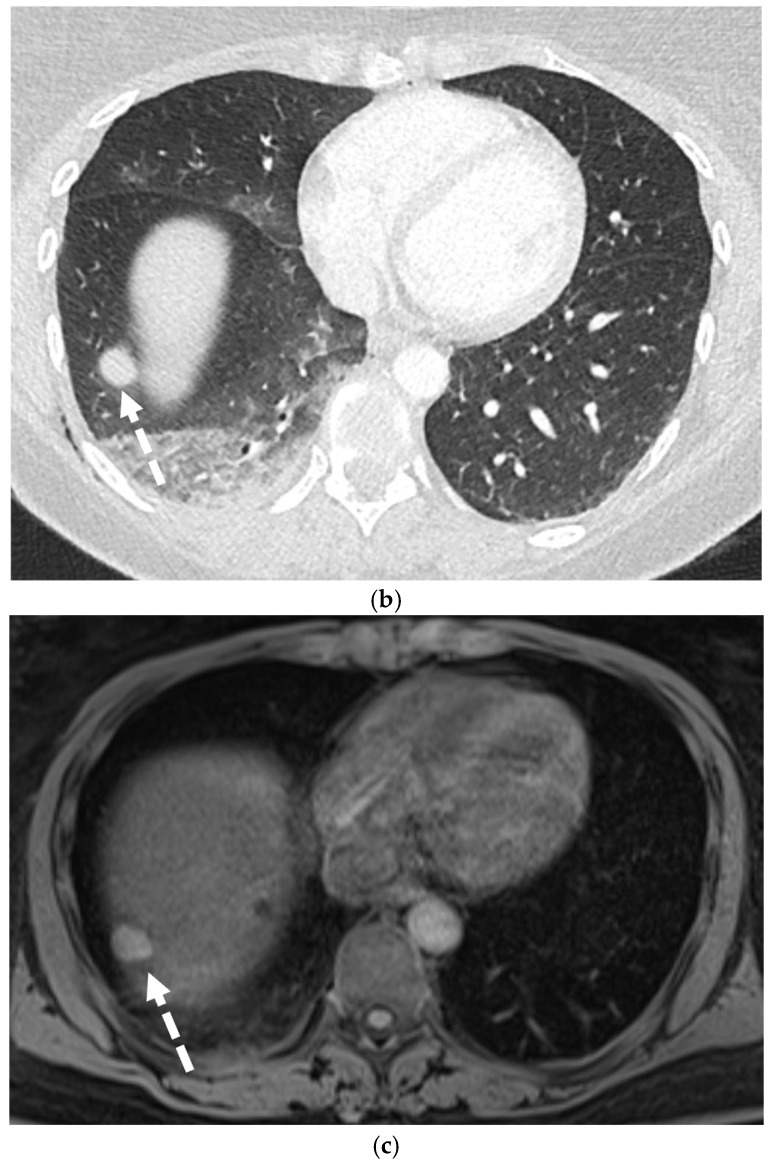
42-year-old female presents with right-sided chest pain. Frontal chest radiograph (**a**) demonstrates large right pneumothorax (white star). Axial CT image in lung window performed after placement of right pleural drain (**b**) shows patchy ground-glass opacities in right-lower lobe and solid nodule (dashed white arrow) along right hemidiaphragm. Axial fat-suppressed T1W MR image (**c**) shows high signal intensity of solid nodule (dashed white arrow) along right hemidiaphragm indicating subacute hemorrhagic/proteinaceous content. These findings are compatible with endometriotic implants with catamenial pneumothorax.

Although rare overall, catamenial pneumothorax is the most frequent cause of recurrent spontaneous pneumothorax in women of reproductive age and therefore warrants specific consideration. As noted in Table 1, while rare, it carries an extremely high risk of recurrence tied to the menstrual cycle. It is defined by the occurrence of pneumothorax in close temporal relationship with menstruation (typically within 72 h of onset). The underlying cause is thoracic endometriosis, where endometrial implants are most often found on the right hemidiaphragm and pleura [75]. While pneumothorax is the most common manifestation, imaging may also reveal pleural effusion, hemothorax, or diaphragmatic nodules. CT is the primary modality for identifying these associated findings. MRI is particularly valuable as it can identify the high T1 signal characteristic of subacute hemorrhage within endometriotic implants on the pleura or diaphragm, which may not be apparent on CT [75,76,77,78,79] (Figure 28).

#### 5.7.2. Electronic-Cigarette or Vaping Use-Associated Lung Injury (EVALI)

**Figure 29:**
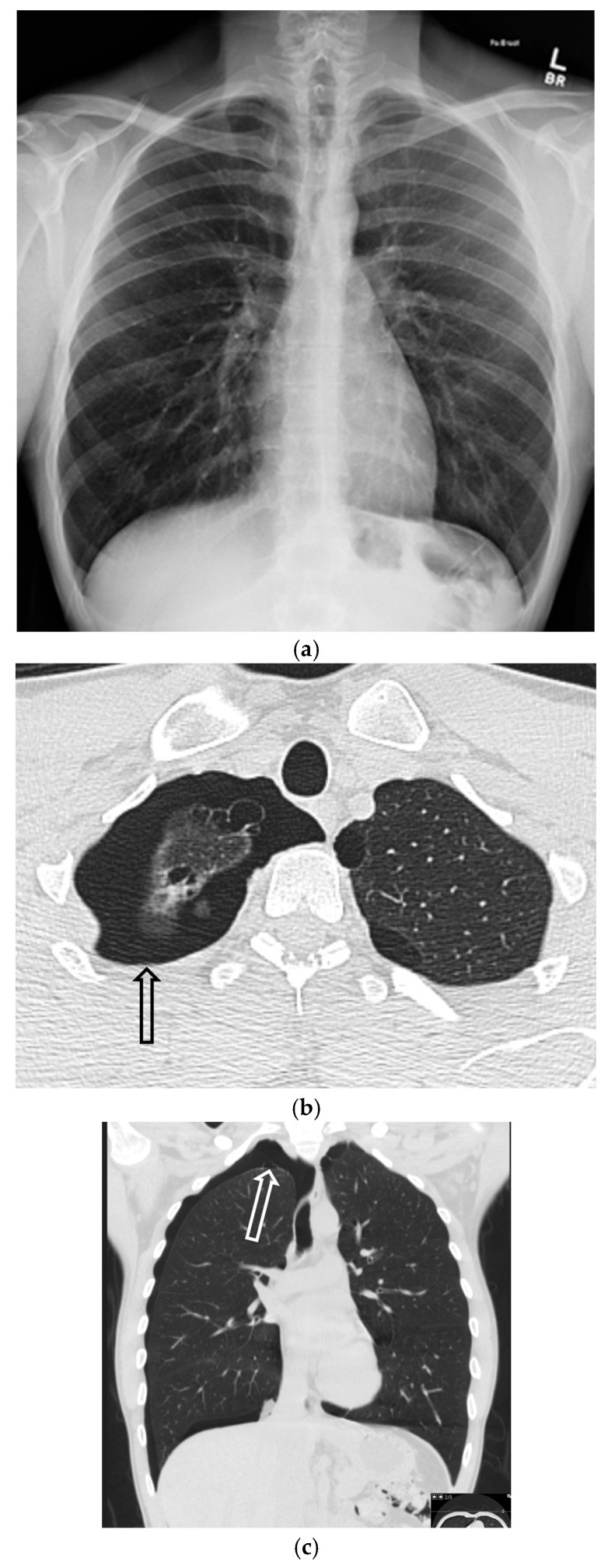

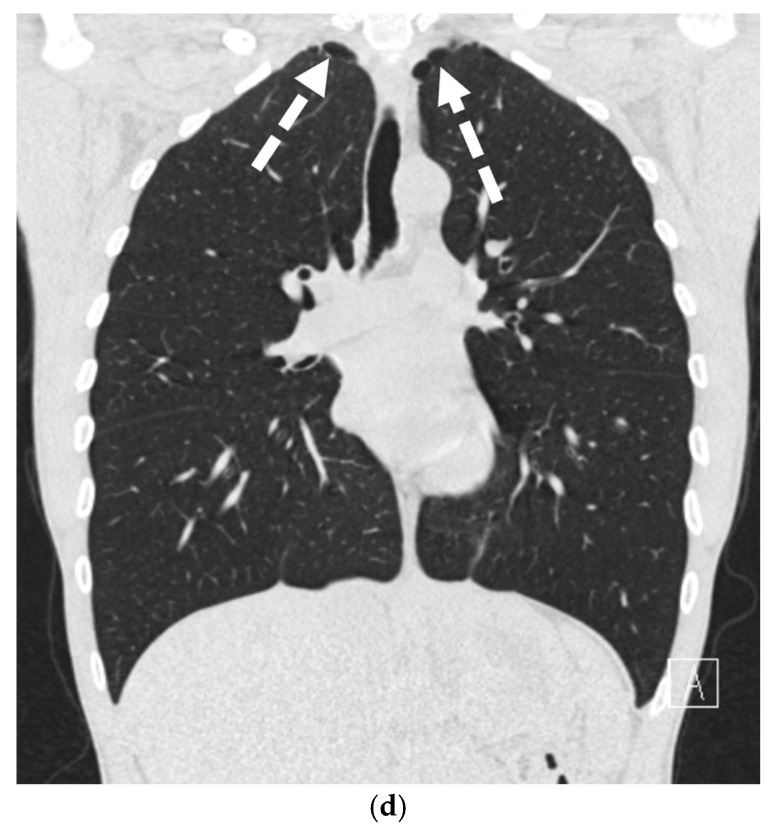
31-year-old male with a history of vaping nicotine-containing substances presents with chest pain. The frontal chest radiograph (**a**) is unremarkable. Axial and coronal CT images in lung window (**b**–**d**) demonstrate small right pneumothorax (open black arrow) as well as biapical paraseptal emphysema and blebs/bullae (dashed white arrows).

Spontaneous pneumothorax is a recognized complication of electronic cigarette or vaping use-associated lung injury (EVALI). The mechanism is not fully elucidated but is thought to involve intense airway inflammation and alveolar damage from inhaled toxins, leading to the formation of subpleural blebs that are prone to rupture. While the underlying CT findings of EVALI are non-specific and can mimic organizing pneumonia or hypersensitivity pneumonitis, the presence of apical paraseptal emphysema and blebs should raise suspicion in this clinical context [80,81] (Figure 29).

#### 5.7.3. Other Acquired and Systemic Causes

Other acquired causes of SP include drug-induced pneumothorax, a rare complication of medications like bleomycin or methotrexate that cause parenchymal fragility [82]. Barotrauma from mechanical ventilation, diving, or altitude changes can lead to alveolar rupture, often via the Macklin effect [83,84,85]. Finally, severe metabolic disturbances, most notably anorexia nervosa, are known to cause emphysema-like changes that increase alveolar fragility from profound malnutrition [86,87].

#### 5.7.4. Neurofibromatosis Type 1 (NF1)

**Figure 30:**
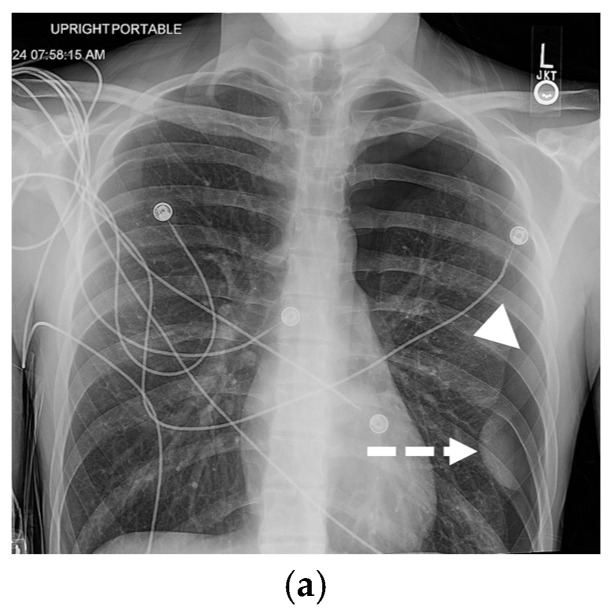

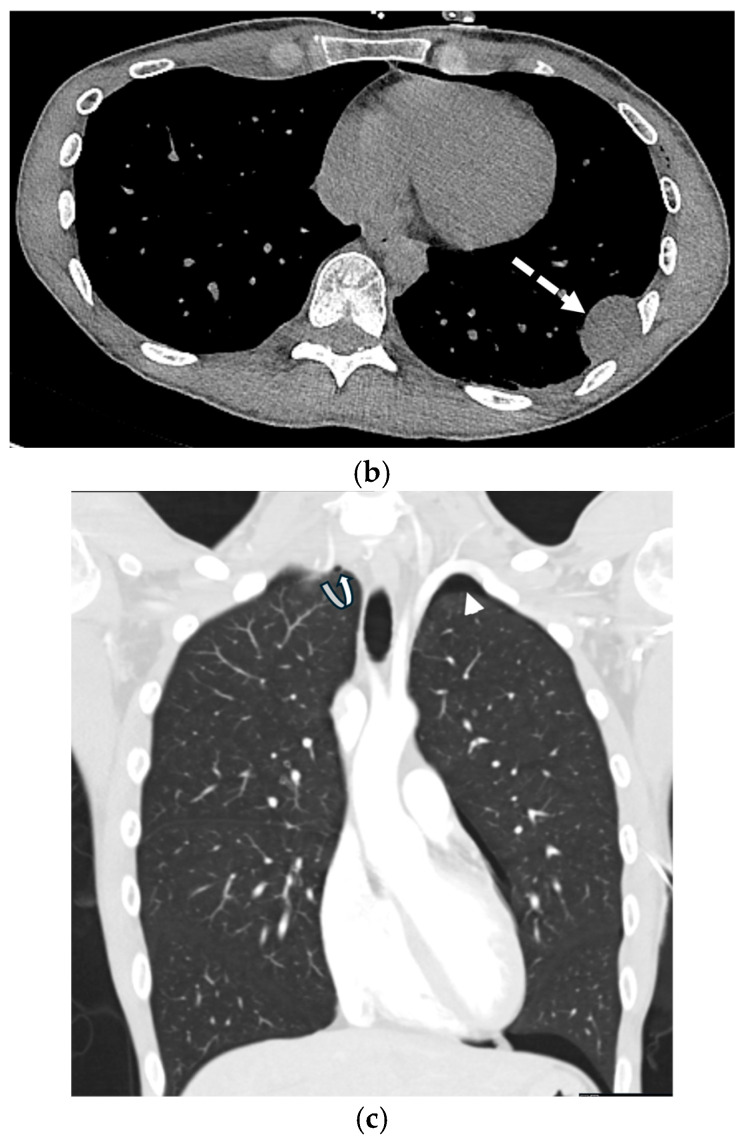
20-year-old male presents with SP. Frontal chest radiograph (**a**) shows moderate left pneumothorax (white arrowhead), along with pleural-based nodular opacity with an incomplete border sign in the inferior left hemithorax (dashed white arrow). Axial CT image in soft tissue window (**b**) demonstrates a well-circumscribed low attenuation lesion in the posterolateral left chest wall (dashed white arrow). Coronal CT image in lung window (**c**) reveals subpleural blebs in the right lung apex (curved white arrow) and small left pneumothorax (white arrowhead), status post-left chest tube placement. A subsequent surgical biopsy confirmed the diagnosis of neurofibroma.

SP occurs in 5–10% of patients with NF1 due to the rupture of apical bullae associated with NF1-related cystic lung disease. The diagnosis should be suspected when a pneumothorax is seen with thoracic stigmata like neurofibromas or ribbon-like rib deformities [17,88,89,90,91,92] (Figure 30).

#### 5.7.5. IgA Vasculitis (Formerly Known as Henoch–Schönlein Purpura (HSP))

**Figure 31:**
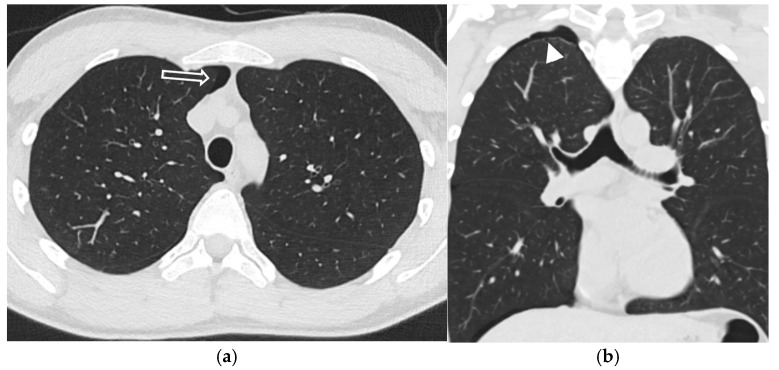
19-year-old male with a history of IgA nephropathy and vasculitis presents with chest pain. Axial and coronal CT images in lung window (**a**,**b**) demonstrate a small right pneumothorax (open white arrow). The coronal image also shows small right apical blebs (white arrowhead).

SP is an exceedingly rare complication of IgA vasculitis (formerly Henoch–Schönlein purpura, HSP), with a proposed mechanism involving pulmonary capillaritis and subsequent subpleural bleb formation [93] (Figure 31).

#### 5.7.6. Loeys–Dietz Syndrome

In Loeys–Dietz syndrome, a genetic connective tissue disorder, SP can be an occasional presenting feature. The diagnosis is suggested by associated vascular findings (arterial tortuosity, aneurysms, dissections) and skeletal abnormalities (pectus deformities, scoliosis) [94,95].

## 6. Practical Considerations: Age-Related Variations

The etiologies of SP vary significantly at the extremes of age. In children and neonates, while over half of cases are primary (PSP), secondary causes related to infections, cystic fibrosis, or asthma are common. Pulmonary interstitial emphysema is a key factor in neonates with respiratory distress syndrome [96,97]. In the elderly, SP is overwhelmingly secondary (SSP), most commonly from COPD, and is associated with significantly higher morbidity and mortality, especially when related to underlying interstitial pneumonia [98].

## 7. Conclusions

Secondary spontaneous pneumothorax (SSP) presents a diagnostic challenge due to its vast array of underlying causes beyond COPD. Identifying the specific etiology is crucial for management and predicting recurrence, making the radiologist’s role vital. While chest radiography is the first step, CT remains superior for detecting subtle pathologies. A thorough understanding of the diverse causes of SSP, paired with appropriate imaging, is essential for improving patient outcomes. Future research should focus on the clinical impact of emerging technologies like photon-counting CT and the integration of genetic testing.

## Figures and Tables

**Table 1:** Etiologies of secondary spontaneous pneumothorax (SSP) stratified by frequency, demographics, and recurrence risk.

Risk Category	Condition	Typical Age of Onset	Sex Predominance	Approximate Incidence/Prevalence	Recurrence Rate
Common (Often the primary differential)	Chronic obstructive pulmonary disease (COPD)	>55 years	Male	Leading cause of SSP (50–70%).	High (30–50%)
	Idiopathic pulmonary fibrosis (IPF)	>60 years	Male	Second most common cause; 2–20% of IPF patients.	High
Occasional (Frequently encountered)	Malignancy (Primary lung or metastatic)	>60 years	Equal	Up to 10% of SSP cases.	High
	Tuberculosis	Any age	Male	~1% of active TB; leading cause in endemic regions.	Moderate
	Cystic fibrosis	Childhood to young adult	Equal	Lifetime risk of 10–20%; annual incidence of up to 3.4% in CF patients.	Very high (>50%)
	Pneumocystis jirovecii pneumonia	Any age (Immunocompromised)	Male	~33% of patients with PJP and pneumatoceles.	High
	Sarcoidosis	20–40 s	Slightly female	~2% of patients; typically late-stage/fibrotic disease.	Moderate to high
	Marfan syndrome	Adolescence to young adult	Equal	4–14% prevalence in affected individuals.	High
	COVID-19 pneumonia	Any age (higher risk in >65)	Male	Incidence: ~1–1.4%.	Variable
Rare (Important but less frequent)	Rheumatoid arthritis (With lung involvement)	30–50 s	Female	Up to 5% of patients with RA-ILD.	Moderate to high
	Lymphangioleiomyomatosis	Reproductive age (20–40 s)	Female	~40% lifetime risk.	Very high (up to 70%)
	Birt–Hogg–Dubé syndrome	30–50 s	Equal	Lifetime risk: 22–41%.	Very high
	Catamenial pneumothorax	Reproductive age (20–40 s)	Female	Most common cause of recurrent SP in reproductive-age women.	Extremely high (recurs with menstrual cycle)
	Ehlers-Danlos syndrome	Childhood to young adult	Equal	High prevalence in vEDS; often a presenting feature.	High
	Pulmonary Langerhans cell histiocytosis	20–40 s	Equal	Pneumothorax is a presenting feature in ~15% of patients; 0.25–0.5% of all SP cases.	High

**Table 2:** Radiological evaluation of pneumothorax: a comparison of modalities.

Modality	Features	Advantages	Disadvantages	Recommended Technique/Protocol
Radiography	Visceral pleural line Lucent peripheral space Deep sulcus sign (supine)	Widely available, low cost, low radiation.	Low sensitivity for small/loculated pneumothorax and underlying causes. Expiratory images may help slightly.	PA and lateral views Optional: Expiratory or lateral decubitus views to increase conspicuity.
Ultrasonography	Absence of “lung sliding” “Lung point” (highly specific) “Barcode sign” (M-mode)	Rapid, bedside, no radiation exposure, good for critically ill/children, excludes pneumothorax well.	Operator dependent, limited view (ribs), poor for detecting underlying cause.	High-frequency linear probe (5–12 MHz) Scan least dependent area of chest wall.
EID-CT	Subtle or loculated pneumothorax Subpleural blebs/bullae Characterizes underlying disease (e.g., ILD, emphysema)	Reference standard for detection and cause, quantification.	Higher radiation exposure/cost, less accessible than radiography or ultrasonography.	Non-contrast thin-slice (e.g., 1–1.25 mm) Reconstruct with high-spatial-frequency (lung) algorithm; MPR essential.
PCD-CT	Improved visualization of subtle findings (e.g., small blebs)	Potential for improved detail, radiation dose reduction.	Limited availability, clinical impact is still evolving.	Protocols are evolving; based on standard non-contrast chest protocols.

Abbreviations: Multiplanar reconstruction (MPR), energy-integrating detector-computed tomography (EID-CT), and photon-counting detector-computed tomography (PCD-CT).

## Data Availability

Not applicable.

## Abbreviations

AIDS	acquired immunodeficiency syndrome
BHDS	Birt–Hogg–Dubé syndrome
CF	cystic fibrosis
COPD	chronic obstructive pulmonary disease
CT	computed tomography
CTA	computed tomography angiography
CXR	chest X-ray
EID	energy-integrating detector
EID-CT	energy-integrating detector-computed tomography
EVALI	electronic-cigarette or vaping product use-associated lung injury
FDG	^18^F-fluorodeoxyglucose
GGO	ground-glass opacity
HCC	hepatocellular carcinoma
HIV	human immunodeficiency virus
HSP	Henoch–Schönlein purpura
IgA	immunoglobulin A
ILD	interstitial lung disease
IPF	idiopathic pulmonary fibrosis
IV	intravenous
LAM	lymphangioleiomyomatosis
LCH	Langerhans cell histiocytosis
LIP	lymphoid interstitial pneumonia
MFS	Marfan’s syndrome
MRI	magnetic resonance imaging
NF1	neurofibromatosis type 1
NSIP	non-specific interstitial pneumonia
PCD	photon-counting detector
PCD-CT	photon-counting detector-computed tomography
PET/CT	positron emission tomography/computed tomography
PJP	pneumocystis jirovecii pneumonia
PLCH	pulmonary Langerhans cell histiocytosis
PSP	primary spontaneous pneumothorax
SP	spontaneous pneumothorax
SPECT/CT	single-photon emission computed tomography/computed tomography
SSP	secondary spontaneous pneumothorax
TSC	tuberous sclerosis complex
vEDS	vascular Ehlers–Danlos syndrome
